# FAM72, Glioblastoma Multiforme (GBM) and Beyond

**DOI:** 10.3390/cancers13051025

**Published:** 2021-03-01

**Authors:** Nguyen Thi Thanh Ho, Chinmay Satish Rahane, Subrata Pramanik, Pok-Son Kim, Arne Kutzner, Klaus Heese

**Affiliations:** 1 Graduate School of Biomedical Science and Engineering, Hanyang University, 222 Wangsimni-ro, Seongdong-gu, Seoul 133-791, Korea; nguyenho1408@hanyang.ac.kr; 2 Maharashtra Institute of Medical Education and Research, Talegaon Dabhade, Maharashtra 410507, India; chinsanity@gmail.com; 3 Institute of Biotechnology, RWTH Aachen University, Worringerweg 3, 52074 Aachen, Germany; s.pramanik@biotec.rwth-aachen.de; 4 Department of Mathematics, Kookmin University, 77 Jeongneung-ro, Seongbuk-gu, Seoul 136-702, Korea; pskim@kookmin.ac.kr; 5 Department of Information Systems, College of Computer Science, Hanyang University, 222 Wangsimni-ro, Seongdong-gu, Seoul 133-791, Korea; kutzner@hanyang.ac.kr

**Keywords:** brain cancer, cell cycle, differentiation, glioblastoma, proliferation, RAS, SRGAP2, stem cell, TP53

## Abstract

**Simple Summary:**

Glioblastoma multiforme (GBM) is a serious and aggressive cancer disease that has not allowed scientists to rest for decades. In this review, we consider the new gene pair |-SRGAP2–FAM72-| and discuss its role in the cell cycle and the possibility of defining new therapeutic approaches for the treatment of GBM and other cancers via this gene pair |-SRGAP2–FAM72-|.

**Abstract:**

Neural stem cells (NSCs) offer great potential for regenerative medicine due to their excellent ability to differentiate into various specialized cell types of the brain. In the central nervous system (CNS), NSC renewal and differentiation are under strict control by the regulation of the pivotal SLIT-ROBO Rho GTPase activating protein 2 (SRGAP2)—Family with sequence similarity 72 (FAM72) master gene (i.e., |-SRGAP2–FAM72-|) via a divergent gene transcription activation mechanism. If the gene transcription control unit (i.e., the intergenic region of the two sub-gene units, SRGAP2 and FAM72) gets out of control, NSCs may transform into cancer stem cells and generate brain tumor cells responsible for brain cancer such as glioblastoma multiforme (GBM). Here, we discuss the surveillance of this |-SRGAP2–FAM72-| master gene and its role in GBM, and also in light of FAM72 for diagnosing various types of cancers outside of the CNS.

## 1. Introduction

**Figure 1:**
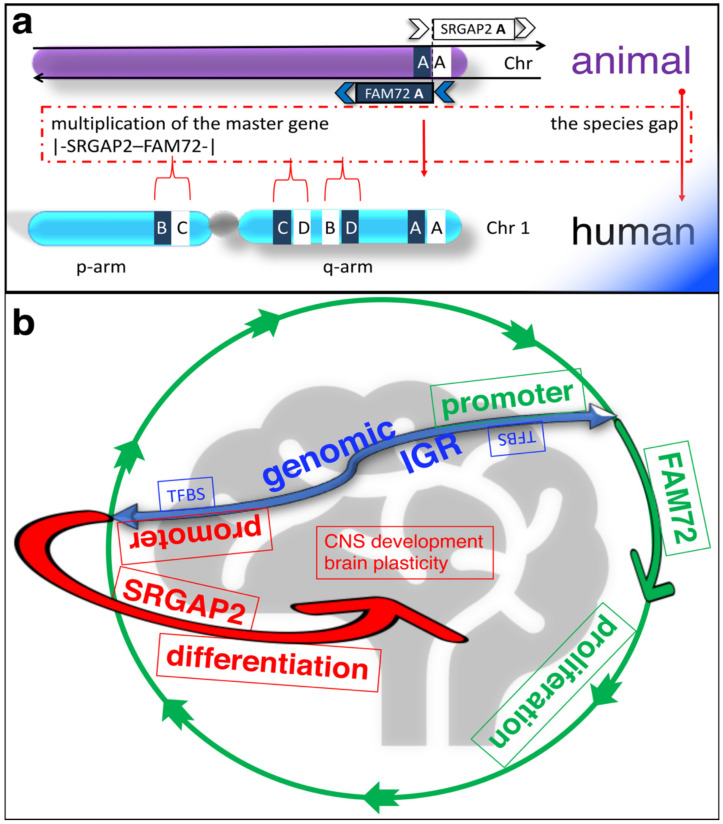
Overview scheme of the |-SRGAP2–FAM72-| master gene expression across the phylogenetic tree. (**a**) While humans express four master genes on chr 1, all other notochord containing vertebrates carry only one such master gene; other species do not show any such master gene, and thus far, no species have been found that show two or three such master genes. FAM72 shows four exons (149 amino acids (aa)), SRGAP2 is composed of 22 exons (1071 aa), and both sub-genes are separated by a 4-kbp intergenic region (IGR). The four paralogous gene pairs A–D are located on opposite strands from one another [1,2,5]. (**b**) Simplified divergent gene transcription paradigm scheme of the novel pivotal |-SRGAP2–FAM72-| master gene in the brain. The |-SRGAP2–FAM72-| master gene resides within a nucleosome-depleted region with the IGR (blue), containing potential transcription factor (TF)-binding sites (BS) (TFBS) between the SRGAP2 (red) and FAM72 (green) genes indicated. Reverse-oriented SRGAP2 (red) and FAM72 (green) genes are expressed from opposite DNA strands [1,8]. The dual IGR promotor controls the two reverse-oriented reciprocal functional-dependent genes FAM72 and SRGAP2, respectively, located on opposite DNA strands. If FAM72 gene is activated by TFs, then the transcription of the SRGAP2 gene is activated until it is actively terminated early and vice versa for neuronal differentiation; accordingly, if FAM72 is in the ‘on’ modus, SRGAP2 is switched off and vice versa [8,9,10,11]. Through this mechanism, FAM72 maintains renewal and proliferation of a critical mass of NSCs during brain development while SRGAP2 promotes escape of the cell cycle fostering neuronal differentiation and brain plasticity [5,8]. This structure represents a novel paradigm for controlling the transcription of divergent genes in regulating NSC gene expression and may allow for novel therapeutic approaches to restore or improve higher cognitive functions and cure cancers (Figure 2).

The human brain is a unique organ that can perform higher cognitive functions and is therefore different from all other species. Its uniqueness is reflected in the expression of four paralog gene pairs |-SRGAP2–FAM72-| (A–D) [1,2]. FAM72 is active in proliferating neural stem cells (NSCs) found in the brain hippocampus [1,2,3,4,5]. There are four specific FAM72 (A–D) paralogs associated with four respective SRGAP2 paralogs on human chromosome 1 (chr 1), but only one such gene pair co-exists as the |-SRGAP2–FAM72-| master gene in all other notochord containing vertebrates (Figure 1a) [1,2,6,7].

## 2. Physiological Function of the |-SRGAP2–FAM72-| Master Gene

Endogenous FAM72 expression has been shown in the hippocampal dentate gyrus [3], where the |-SRGAP2–FAM72-| master-gene regulates NSC renewal, neurogenesis and brain plasticity [4,5]. Here, the |-SRGAP2–FAM72-| master-gene is under a divergent gene expression control (Figure 1b) [5,8]. Thus, FAM72 expression is switched on to promote NSC renewal and proliferation and is switched off (concomitantly SRGAP2 is switched on) to foster differentiation, neuritogenesis, synaptic plasticity, and brain development (Figure 1b) [4,5,8,16,17,18,19,20,21]. However, this divergent expression paradigm is currently restricted to neural tissue [5,8] and apoptosis is induced if it gets out of control (i.e., neuronal expression of FAM72 forces reentry into the cell cycle) [3].

## 3. Pathophysiological Function of the |-SRGAP2–FAM72-| Master Gene—FAM72 Expression in Various Types of Cancer

Early studies revealed that FAM72 was overexpressed outside the nervous system in various types of cancer with the protein kinase C signaling pathway activated in neuroblastoma and breast adenocarcinoma (e.g., MCF-7 and MDA-MB-231 cells) [3] and uracil DNA glycosylase-2 as a binding partner in malignant colon cancers [22]. FAM72B was identified as a member of a 7-gene signature in prostate cancer [23], and it was also upregulated in multiple non-neuronal tissues as well [12]. FAM72B, C, and D were also among the highly upregulated genes in B-cell lymphoma [24]. Recently, FAM72D has been identified as a specific proliferation marker in multiple myelomas [25]. Moreover, we reported increased mean expression of FAM72 paralogs across human tumors compared to control tissues, except in cases of skin cutaneous melanoma, kidney chromophobes, and pheochromocytomas. This indicates that neuronal FAM72 paralogs are being expressed in non-neuronal proliferating tumor tissue cells [12].

### 3.1. The |-SRGAP2–FAM72-| Master Gene in Brain Cancer

**Figure 2:**
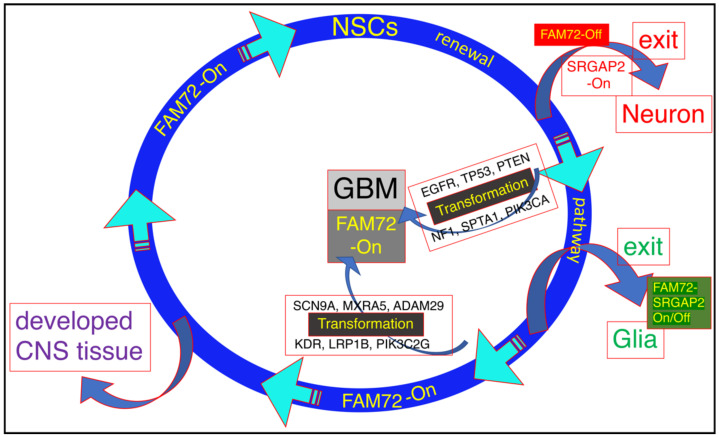
Overview of the |-SRGAP2–FAM72-| master gene expression in GBM [12]. As long as FAM72 remains in the on modus, NSCs keep proliferating. For neuronal differentiation and brain plasticity, FAM72 needs to be switched off to allow SRGAP2 activation and brain development. The activity of the |-SRGAP2–FAM72-| master gene expression during glia cell differentiation is less clear. Since glia cells have the capacity to proliferate, FAM72 might be switched on or off [13,14,15]. Eventually, mutations in GBM-specific driver genes: epidermal growth factor receptor (EGFR), tumor protein p53 (TP53), phosphatase and tensin homolog (PTEN), neurofibromin 1 (NF1), spectrin alpha, erythrocytic 1 (SPTA1) and phosphatidylinositol-4,5-bisphosphate 3-kinase catalytic subunit alpha (PIK3CA) or sodium voltage-gated channel alpha subunit 9 (SCN9A), matrix remodeling associated 5 (MXRA5), a disintegrin and metalloprotease domain 29 (ADAM29), kinase insert domain receptor (KDR), phosphatidylinositol-4-phosphate 3-kinase catalytic subunit type 2 gamma (PIK3C2G), and low-density lipoprotein receptor related protein 1B (LRP1B) induce NSC transformation into cancer stem cells (CSCs) while FAM72 is still in the on modus [12].

Previously, we correlated FAM72 (A–D) mRNA expression z-scores and highly mutated protooncogenes as well as unique mutated genes in deceased GBM patients. mRNA expression and mutation data for GBM was retrieved from cBioportal. Normalized mRNA expression z-score data were computed for all GBM samples and the data for FAM72 (A–D) paralogs were grouped in bins with a size of 0.7 z-score units and correlated with genes showing high numbers of tissue-specific gene mutations. Linear regression was determined first between the FAM72 (A–D) paralogs and then between all available genes in the GBM study, then visualized using online Python-based Bokeh software. A complex brain-specific gene-mutation signature: EGFR, TP53, PTEN, NF1, SPTA1, PIK3CA or SCN9A, MXRA5, ADAM29, KDR, PIK3C2G, and LRP1B was identified that correlated with high FAM72 expression and may lead to cell cycle activation, cell transformation, and cell proliferation. This led to the identification of several pivotal driver genes responsible for the transformation of NSCs into CSCs and GBM (Figure 2) [12].

On the other hand, the partner gene SRGAP2 showed no change in expression in GBM. SRGAP2 is reported to be a tumor suppressor [26], and its expression is usually induced when FAM72 expression is blocked. NSCs stop proliferating during neural differentiation and neuronal synaptogenesis [4,5,8,16,17,18,19,20,21], but may lead to apoptosis in non-neuronal tissue or proliferating cancerous cells [3,5]. Genomic rearrangements causing loss of physiological functions of SRGAP2 may enhance cell motility and metastasis [26].

### 3.2. The |-SRGAP2–FAM72-| Master Gene in Other Cancerous Tissues

**Figure 3:**
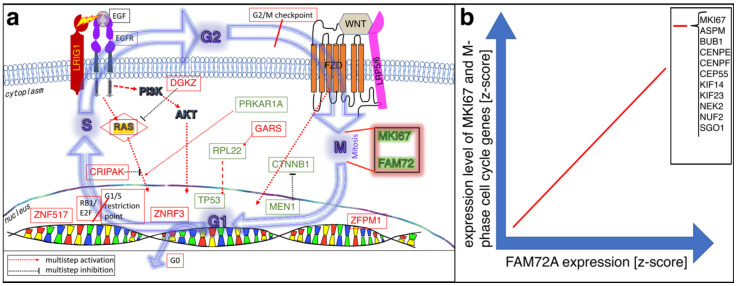
Experimental evidence-based schematic illustration of FAM72 and MKI67 co-activation in adrenocortical carcinoma (ACC). (**a**) Mutations in ACC-specific driver proto-onco- or tumor-suppressor-genes (red and green color) push the cell through the cell cycle and mediate MKI67 as well as FAM72 expression during the M-phase. Red-colored proto-oncogenes (or tumor-suppressor genes) are from Rahane et al. [28], while green-colored proto-oncogenes (or tumor-suppressor genes) are from Zheng et al. [29]; additional ACC-specific cell cycle information are from Assié et al. [30], Lippert et al. [31], and Pereira et al. [32]. Tumor suppressor LRIG1 interferes with EGFR signaling and might be a druggable protein of primary interest [33,34,35]. (**b**) Schematic illustration of mRNA expression correlation between FAM72A on the one hand and M-phase cell cycle genes, including MKI67, on the other hand. FAM72A expression correlates with the expression of cell cycle phase-specific genes across various human cancer tissue. Genes specifically associated with the late G2- to M-phase of the cell cycle, including ASPM, BUB1, CENPE, CENPF, CEP55, KIF14, KIF23, NEK2, NUF2, and SGO1 (ASPM, BUB1, CEP55, KIF14, KIF23, and NEK2 are involved either with spindle formation or with regulation; CENPE, CENPF, NUF2, and SGO1 are involved in the centromere-kinetochore complex) [12,28]. ASPM, Assembly factor for spindle microtubules; BUB1, Budding uninhibited by benzimidazoles 1 mitotic checkpoint serine/threonine kinase; CENPE, Centromere protein E; CENPF, Centromere protein F; CEP55, Centrosomal protein 55; CRIPAK, Cysteine-rich p21-activated protein kinase 1 inhibitor; CTNNB1, Catenin beta 1; DGKZ, Diacylglycerol kinase zeta; FZD, Frizzleds; GARS1, Glycyl-tRNA synthetase 1; KIF14/23, Kinesin family member 14/23; LRIG1, Leucine rich repeats and immunoglobulin-like domains 1; NEK2, Never in mitosis gene a-related kinase 2; NUF2, NUF2 component of NDC80 kinetochore complex; RPL22, Ribosomal protein L22; PRKAR1A, Protein kinase cAMP-dependent type I regulatory subunit alpha; RAS, Rat sarcoma; SGO1, Shugoshin 1; WNT1, Wingless and Int-1 family member 1; ZFPM1, Zinc finger protein, friend of GATA family member 1; ZNF517, Zinc finger protein 517; ZNRF3, Zinc and ring finger 3.

Our recent large-scale tissue analysis demonstrated that the Ki-67 gene (MKI67) and FAM72 paralogs are co-expressed in proliferating cells in NSCs and also outside neuronal tissue (i.e., in cancer cells across various tissues) (Figure 3, Supplementary Materials Figure S1). FAM72 does not appear to be a protooncogene and the reciprocal expression dependency of SRGAP2 and FAM72 seems to be limited to the nervous system. Outside the nervous system, FAM72 expression appears to be induced by a different cancer-causing oncogene [12,27,28]. 

### 3.3. FAM72 in Adrenocortical Carcinoma

Our understanding of the molecular mechanism driving ACC has advanced. Alterations in the components of the WNT1/β-catenin, EGFR, and TP53 pathways are prominent markers in ACC [29,30,31,32]. CTNNB1 and TP53 mutations are mutually exclusive in aggressive adrenal cancers [36]. Activating mutations in CTNNB1 have been observed in approximately 25% of adrenocortical cancers [37]. TP53 mutations have been observed in more than 50% of child patients, but only in 4% of adult patients of ACC [38,39]. 

Recently, we identified a complex novel ACC-specific gene signature: CRIPAK, DGKZ, GARS1, LRIG1, ZFPM1, and ZNF517, which was significantly, specifically, and most repeatedly mutated in ACC and correlated with high FAM72 expression (Figure 3) [28]. This gene set is involved in tumor suppression and cellular proliferation and thus could be useful for the prognosis and development of therapeutic approaches for the treatment of ACC. 

Experimental evidence indicates that EGFR signaling is an anchor body through which proliferative pathways can be initiated and most of the proto-oncogenes in ACC act downstream of EGFR. Moreover, in ACC, LRIG1 mutations would cause a continuous expression of the EGFR signaling cascade, thereby causing cellular proliferation. Inhibition of EGFR via tumor suppressor LRIG1 is thus a key step in regulating (either partially or fully) the consequent signaling cascades. Mutations in GARS1 also serve to increase proliferation via a cascade that is, however, independent of the phosphoinositide-3-kinase (PI3K)/mitogen-activated protein kinase 1 (MAPK1)/WNT1 signaling pathways. Mutations in our novel gene set thus appear to be more influential in ACC tumorigenesis than those described in earlier studies and could serve as a powerful therapeutic target [28,29].

## 4. FAM72 and Its Role in the Cell Cycle 

### 4.1. FAM72 in the M-Phase of the Cell Cycle

FAM72 (A–D) is highly expressed when promoting NSC and cancer cell proliferation and are present in the G2/M phase of the cell cycle [2,5,12,28]. It has been shown that knock-down of FAM72A in NSCs blocks cell proliferation and causes cell differentiation [4]. In line with this, FAM72B knockdown experiments showed that cell proliferation was reduced in human fibroblasts [40], suggesting that FAM72B also has a common role in promoting cell proliferation, similar to the other FAM72 members. Cell cycle specific expression analysis revealed that FAM72 (A–D) activity occurred particularly during the G2/M-phase, but not during the G1/S-phase (Figure 3b) [12,28].

**Figure 4:**
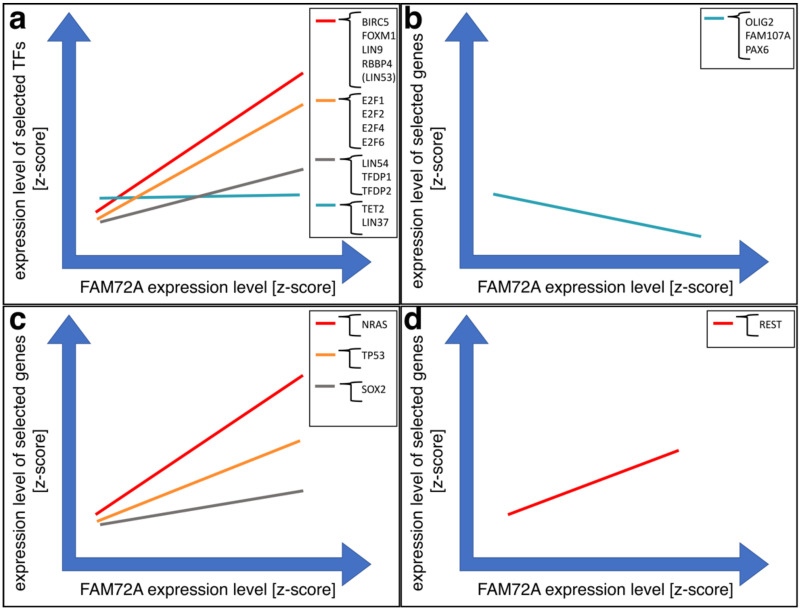
Schematic illustration of mRNA expression correlation of FAM72A compared with several other GBM-relevant genes, including E2Fx TFs. (**a**) FAM72A expression correlates with the expression of selected genes and TFs (Supplementary Materials Figures S2–S17). (**b**) FAM72A expression does not correlate with the expression of OLIG2, FAM107A nor with PAX6 (Supplementary Materials Figures S7, S12, and S13). (**c**) FAM72A expression correlates with neuroblastoma rat sarcoma proto-oncogene (NRAS), TP53, and weakly with sex determining region Y (SRY) box transcription factor 2 (SOX2) in glioma (Supplementary Materials Figures S18–S20). (**d**) FAM72A expression correlates with RE1 silencing transcription factor (REST) in glioma (Supplementary Materials Figure S21).

NSC or cancer cell fate is determined based on specific E2 factor transcription factor E2Fx TFs (x = 1, 2, 3, 4 and 6, i.e., E2F1, E2F2, E2F3, E2F4, E2F6 such as E2F6 in a complex with transcription factor dimerization partner 1 [TFDP1]) bound to the promoter within the IGR of the |-SRGAP2–FAM72-| master gene. We found that FAM72 expression correlates with the expression of a baculoviral inhibitor of apoptosis protein (IAP) repeat (BIR)-containing 5 (BIRC5, also known as survivin), Forkhead box M1 (FOXM1), LIN9, LIN54 (partially), and retinoblastoma binding protein 4 (RBBP4) (Lin53, partially) and also with pivotal E2Fx TFs in various cancer tissues including brain glioma. Other genes showed either weak (TFDP1 and TFDP2) or no correlation (oligodendrocyte marker OLIG2, tumor suppressor family with sequence similarity 107 member A [FAM107A]), paired box protein Pax-6 (PAX6), and ten eleven translocation protein 2 (tet methylcytosine dioxygenases 2, TET2), and LIN37) (Figure 4, Supplementary Materials Figures S2–S16).

### 4.2. FAM72 in the G0 Stage of the Cell Cycle

Some studies showed that retinoblastoma transcriptional corepressor 1 (RB1) may cause the cell to go into the G0 phase with different cell fates: Quiescent G0 with reversible return option to reenter the cell cycle for proliferation, post-mitotic G0 with irreversible cell differentiation, or cell senescence G0, eventually leading to apoptosis [41,42]. Our data suggest that the |-SRGAP2–FAM72-| master gene induces the RB1 pathway (eventually via TP53 acetylation) to push cells into the G0 stage concomitantly with SRGAP2 expression, supporting neural survival and stabilizing a neuronal phenotype at stage G0 [8].

As we reported recently, the dual IGR promoter has an important role in regulating divergent gene transcription of both directions of the |-SRGAP2–FAM72-| master gene [8]. In the context of rat PC12 cells (a well-known neuronal cell model to study neurogenesis [8,43,44,45,46,47,48]), Fam72a expression (in proliferating PC12 cells stimulated by the mitogen Egf) or Srgap2 expression (in differentiating PC12 cells stimulated by nerve growth factor (Ngf)) was enhanced upon growth factor (Ngf or Egf)-mediated stimulation. Strikingly, under serum-withdrawal-induced stress and bi-directional IGR control, Egf-stimulated PC12 cells were kept alive for a long period of time with Fam72a expression, while Ngf-stimulated PC12 cells remained in a G0 stage co-expressing Srgap2 and Fam72a without proliferation [5,8].

### 4.3. Governance of FAM72 Expression: The IGR and Its TFBSs

**Figure 5:**
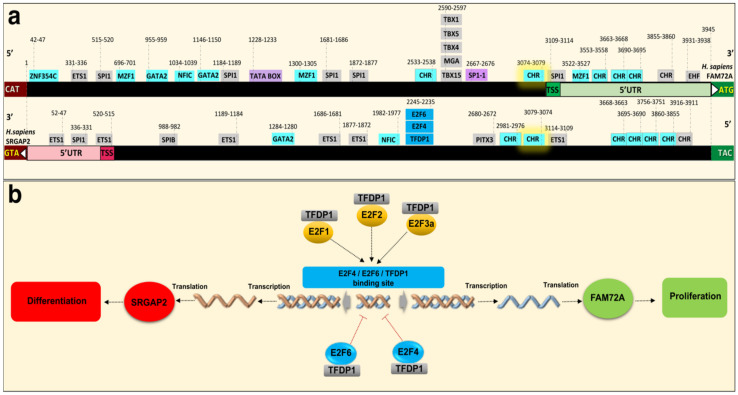
Integrated diagram for putative TFBSs in the intergenic region IGR between the transcription start sites (TSS) of FAM72A and SRGAP2, using the Ensembl and JASPAR databases, and the effect on the cell cycle. (**a**) Putative TFBSs on the IGR between SRGAP2 and FAM72A coding sequences in *Homo sapiens*. Multiple TFBSs are present for binding of the TFs GATA2, SPI1, MZF1, EGR1, SP1, and E2Fx (x = 1, 2, 3, 4 and 6). The open reading frames (ORFs) for FAM72A and SRGAP2 are indicated on the right and left sides, respectively. TFs that are common between FAM72 (A–D) and selected M-phase cell cycle genes are in pale blue. Investigation of the potential TFBSs on the IGR shows that FAM72A is a cell cycle gene particularly active in mitosis and under control of the DREAM and MMB-FOXM1 complexes acting on the CHR BS to regulate |-SRGAP2–FAM72A-|. The DREAM complex is composed of TFDP1, RBL2, or RBL1, the repressor E2F TF E2F4 or E2F5 and the MuvB core complex (containing LIN9, LIN37, LIN52, RBBP4 (LIN53), LIN54). The MMB–FOXM1 complex is composed of the MuvB complex (dissociated from the DREAM complex), MYBL2, and FOXM1. Notably, the CHR site (pale blue) located next to the TSS of the FAM72A gene has the highest potential to be targeted for driving FAM72A gene expression. (**b**) The crucial E2F4/E2F6/TFDP1 BS in cell fate decision. The consensus E2F4/E2F6/TFDP1 BS within the IGR could become occupied by an E2Fx family member depending on cell demand during specific cell phase stages and may be crucial for cell fate decision to activate either FAM72A (for cell proliferation and renewal) or SRGAP2 (for neural differentiation). Chr, chromosome; CHR, Cell cycle gene homology region; E2F1/2/3/4/6, E2 factor TF 1/2/3/4/6; EGR1, early growth response 1; EHF, ETS homologous factor; ETS1, E26 transformation specific proto-oncogene 1; GATA2, GATA binding protein 2; MGA, MAX dimerization protein; MZF1, Myeloid zinc finger 1; NFIC, Nuclear factor I C; SP1-1, Specificity protein 1 TFBS 1; SPI1, Spleen focus forming virus proviral integration oncogene 1; TBX15/TBX1/TBX4, T-box TFBS 15/1/4; TFDP1, TF dimerization partner 1; ZNF345C, Zinc finger protein 345C.

A comparative genome analysis of the IGR (located between the SRGAP2 and FAM72 genes within the |-SRGAP2–FAM72-| master gene on the one hand and the gene promoters of several G2/M-phase-specific cell cycle genes on the other hand) revealed potential common regulatory elements (i.e., common TFBSs), driving the expression of those cell cycle genes and FAM72 to promote and maintain cell proliferation (Figure 3b, Figure 4 and Figure 5a,b). We found that many genes with increased expression during the late G2/M-phase of the cell cycle including all human FAM72 paralogs shared the same TFBS motifs for GATA binding protein 2 (GATA2) [12], E2F4, E2F6, and TFDP1 (Figure 4 and Figure 5a,b). This indicates that their expression is co-regulated in concert with the FAM72 paralogs and implies a common temporal and spatial function, particularly fostering cell proliferation, eventually associated with the RAS signaling pathway [49,50,51,52,53]. 

Additional comparative genome analysis between the FAM72 and MKI67 gene promoters also revealed common potential TFBSs for the TFs GATA2, E26 transformation specific proto-oncogene 1 (ETS1), myeloid zinc finger 1 (MZF1), and nuclear factor I C (NFIC), zinc finger protein 345C (ZNF354C) (Figure 3b and Figure 5a) [12].

To further understand the mechanism of IGR-controlled |-SRGAP2–FAM72-| master gene expression, we performed bioinformatic analysis of TFBSs on the IGR (Figure 5a) [54]. The predicted TFBSs appear to partly explain our questions raised based on their ability to control the cell cycle and transcription regulation of this |-SRGAP2–FAM72-| master gene pair via its IGR. Specifically, we discovered E2F4, E2F6, and TFDP1 TFBSs present on the IGRs of the |-SRGAP2–FAM72A-|, |-SRGAP2C–FAM72B-|, |-SRGAP2D–FAM72C-|, and |-SRGAP2B–FAM72D-| gene pairs (Figure 5a,b). This indicates the participation of a heterodimeric E2Fx/TFDP1 complex, which may contribute to the divergent gene transcription control of FAM72A and SRGAP2, respectively (Figure 5a,b). The E2Fx family is known to consist of TF members, which all play important roles in the cell cycle control. The E2F4/E2F6/TFDP1 predicted sites on the IGR are assumed as binding sites for E2Fx family members with both gene activation or repression abilities [55,56,57]. Interchangeable roles of E2Fx family members were revealed by a comprehensive ChIP analysis of E2F1 (e.g., E2F1-3a activators), E2F4 (e.g., E2F4-5 canonical repressors), and E2F6 (e.g., E2F6-8 atypical repressors) in normal and tumor cells [55], while loss of one E2F member could cause a function compensation by the other E2Fs to ensure cell cycle operation [58,59]. Specifically, E2F6 encodes a member of a family of TFs that plays a crucial role in the control of the cell cycle, of which the protein lacks the transactivation and tumor suppressor protein association domains found in other E2Fx family members, and it contains a modular suppression domain that functions in the inhibition of transcription. It interacts in a complex with chromatin modifying factors. Moreover, TFDP1 encodes a member of a family of TFs that heterodimerize with E2Fx proteins to enhance their DNA-binding activity and promote transcription from E2Fx target genes. The encoded protein functions as part of this complex to control the transcriptional activity of numerous genes involved in cell cycle progression from G1 to the S phase.

In the CNS, E2Fx TFs such as E2F1, E2F2, E2F3, and E2F4, along with the pocket proteins (PPs including RB1, RB-like pocket proteins RBL1 (p107) and RBL2 (p130)), regulate NSC self-renewal via pivotal genes including SOX2, PAX6, fibroblast growth factor 2 (FGF2), distal-less homeobox 1 and 2 (DLX1, DLX2), neogenin 1 (NEO1), and neuropilin 1 (NRP1) as well as the Notch and sonic hedgehog (SHH) pathways [60,61,62,63,64,65,66,67,68,69,70]. Interestingly, E2F4 establishes a proper cell fate both in conjunction with or without RB1 [71,72]. 

Detailed spatiotemporal expression analysis of E2Fx TFs unraveled specific E2Fx activators (E2F3A) and canonical (E2F4) and atypical (E2F8) E2Fx TF repressors during the cell cycle [73]. An orchestrated accumulation of different E2Fx TF combinations control gene expression in proliferating (E2F3A-8-4) and differentiating (E2F3A-4) cells. The sequential nuclear accumulation and disappearance of E2F3A, E2F8, and E2F4 form an E2F module used to drive waves of activation and repression that support cell-cycle-dependent oscillations in gene expression necessary for cell proliferation and cell divisions. Another E2Fx TF module composed of E2F3A and E2F4 is used to extinguish cell-cycle-dependent gene expression in cells programmed to exit the cell cycle and differentiate. With an activity in the G2 phase and a TFBS within the IGR, E2F4 seems to be among the pivotal TFs controlling the |-SRGAP2–FAM72-| master gene (Figure 5). 

Since E2F4 may have both functions of gene activation and repression, we assume that E2F4 could be the key that could repress FAM72A and support SRGAP2 expression during differentiation. This is consistent with the finding that E2F4 permanently accumulates in the nucleus of differentiating and differentiated cells [73].

With these important discoveries about the |-SRGAP2–FAM72-| master gene and its regulatory role regarding cell fate decision [5,8], we looked for partners to cooperate with these two genes. Among the possible candidates, E2Fx TFs and their regulatory partners, the PPs RB1, RBL1 and RBL2, are widespread and dynamic epigenetic stem cell regulators [69]. The E2Fx consensus TFBS on the IGR shows an ability to interact with various E2Fx TFs, which in turn, can bind to the IGR and govern FAM72 as well as SRGAP2 expression (Figure 5). The RB1 and E2Fx TFs make complexes called RB-E2Fx, which cooperate with a protein complex called DREAM (dimerization partner (DP), RB-like, E2F and multi-vulval class B (MuvB)), repressing G1/S cell cycle genes to move the cell cycle forward to the G2/M phase [74,75,76]. The conserved human DREAM complex thus has been described as an important master regulator of cell cycle genes with a decisive role in coordinating cell cycle progression [75,76,77,78,79,80,81].

The DREAM complex comprises TFDP1, RBL1, or RBL2, the repressor E2Fx TFs E2F4 or E2F5 and the MuvB core complex (containing LIN9, LIN37, LIN52, RBBP4 (also known as LIN53), LIN54). 

LIN9, a component of the DREAM core complex, encodes a tumor suppressor protein that inhibits DNA synthesis and oncogenic transformation through association with the RB1 protein. It also interacts with a complex of other cell cycle regulators to repress cell cycle-dependent gene expression in non-dividing cells [82].

RBBP4 is a chromatin remodeling factor that encodes a ubiquitously expressed nuclear protein that belongs to a highly conserved subfamily of WD-repeat proteins [83,84]. It is involved in histone (de-) acetylation and chromatin assembly and remodeling. RBBP4 is also part of co-repressor complexes, which are integral components of transcriptional silencing. It is found among several cellular proteins that bind directly to RB1 to regulate cell proliferation and also seems to be involved in transcriptional repression of E2Fx-responsive genes [85,86].

As an integral subunit of the DREAM complex, LIN54 is a pivotal regulator of cell cycle genes, which binds to the cell division control 2 (CDC2) promoter for cell cycle progression [87].

**Figure 6:**
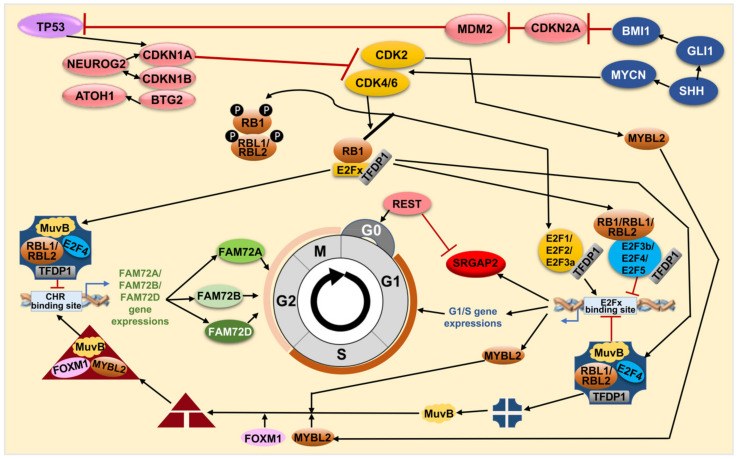
FAM72 paralog-specific cell cycle signaling mediated by various TFs. DREAM (blue rectangle) is composed of TFDP1, RBL1 or RBL2, the E2Fx TFs E2F4, or E2F5 and the MuvB core complex (consists of LIN9, LIN37, LIN52, RBBP4 (LIN53), LIN54). During quiescence/G0 and early G1 phases of the cell cycle, DREAM represses cell cycle gene expression. When these G0/G1 stages end, DREAM gets inactivated so that the MuvB complex dissociates away to form a new complex with the MYBL2 and FOXM1 called the MMB-FOXM1 complex (red triangle, MuvB, MYBL2 and FOXM1). This new complex promotes late cell cycle gene expression and is required to pass through the G2/M phases [80]. At the end of the M-phase, REST inhibits neuronal gene expression (such as SRGAP2) to allow re-entry into a new cycle, thus maintaining NSC renewal and FAM72 expression. Once it receives a neurogenic signal, REST is degraded, FAM72 expression is blocked, and SGRAP2 expression is initiated for neuronal differentiation. ATOH1, Atonal basic helix-loop-helix (bHLH) TF 1; BMI1, B cell-specific Moloney murine leukemia virus integration site 1; BTG2, B-cell translocation gene 2;CDK2/4/6, Cyclin dependent kinase 2/4/6; CDKN1A/1B/2A, Cyclin dependent kinase inhibitor 1A/1B/2A; FOXM1, Forkhead box M1; GLI1, Glioma-associated oncogene family zinc finger 1; MDM2, Murine double minute 2; MuvB, Multi-vulval class B complex; MYBL2, v-myb avian myeloblastosis viral oncogene homolog-like 2; MYCN, v-myc avian myelocytomatosis viral oncogene neuroblastoma derived; NEUROG2, Neurogenin 2; REST, Transcriptional repressor RE1 silencing transcription factor; SHH, Sonic hedgehog signaling molecule.

Previously, we described FAM72 (A–D) expression specifically during the G2/M phase [12,28]. Other scientists have verified that the DREAM and MMB-FOXM1 complexes can bind genomic cell cycle gene homology region (CHR) motifs, suggesting that DREAM and MMB-FOXM1 are crucially involved in regulating FAM72 (A–D) expression during the G2/M phase (Figure 5 and Figure 6) [12,75,76,88]. Indeed, the DREAM complex was verified to bind to the FAM72 promoter, most probably via the CHR BS on the IGR. Notably, the CHR element is conserved on the IGR across all FAM72 (A–D) (Figure 5a) [75,76]. Genome-wide association studies and experimental validation have verified FAM72D as a G2/M cell cycle gene modulated by the DREAM and MMB-FOXM1 complexes [75,76]. These complexes bind and regulate FAM72D through a CHR BS on the IGR.

The DREAM complex interacts with the CHR element and E2Fx TFBSs to inhibit G1/S cell cycle gene expression until MuvB dissociates away to associate with MMB-FOXM1 to push the cell cycle into the G2/M phase [75,76]. During quiescence and early G1 phase of the cell cycle, the DREAM–MuvB complex represses cell cycle-promoting gene expression. When the stages end, it becomes deactivated, while the MuvB complex dissociates away to associate with v-myb avian myeloblastosis viral oncogene homolog-like 2 (MYBL2) and FOXM1, forming the MMB–FOXM1 complex. This new complex promotes late cell cycle gene expression and is required to pass through the G2/M phases [80]. FOXM1 gets phosphorylated during the M phase and regulates the expression of several cell cycle genes such as cyclin B1 (CCNB1) and cyclin D1 (CCND1). It is a crucial TF also found in fostering GBM development and progression by regulating key factors involved in cell proliferation, epithelial to mesenchymal transition (EMT), invasion, angiogenesis, and upregulating WNT1/β-catenin signaling [89].

The cell cycle promoting regulation indeed comes from the interaction between the FOXM1 protein—a part of the MMB-FOXM1 complex—and the FAM72A [75,76,78,81], FAM72B [40,75,76,78,81], and FAM72D [25,75,76,78,81] promotors, confirming that all FAM72 (A–D) paralogs are regulated by this pathway during the G2/M phase in proliferating cells (i.e., NSCs and cancer cells). Since the FAM72 function may contribute to the mitotic spindle or the kinetochore-centromere complex formations and activities, loss of MMB-FOXM1 or FAM72 (A–D) function may cause spindle assembly chaos and mitotic catastrophe (Figure 6) [12,25].

Taken together, FAM72A, FAM72B, and FAM72D might be regulated by the DREAM complex as well as the RB-E2F3b/4/5 complex to be suppressed for a while by interacting with the putative E2F4/E2F6/TFDP1 TFBS, until the E2F1/2/3a/activators promote essential G1/S gene expressions and thereby foster cell cycle progression into G2/M phases and FAM72 activation via the MMB–FOXM1 complex. Thus, cell cycle progression and control depend on targeting the genomic E2F4/E2F6/TFDP1 TFBS (for G1/S phase) and the CHR motif (for G2/M phase) on the IGR with pivotal regulators involved such as DREAM, the RB family members-E2Fx-, and the MMB-FOXM1 complexes.

### 4.4. FAM72 Expression and the RE1 Silencing Transcription Factor

REST was initially identified as a transcriptional repressor that represses neuronal genes in non-neuronal tissues [90,91]. However, depending on the cellular context, this gene can act as either an oncogene or a tumor suppressor, and its specific role in glioma remains controversial [92,93]. The encoded protein is a member of the Kruppel-type zinc finger transcription factor family. It represses transcription by binding a DNA sequence element called the neuron-restrictive silencer element [94,95]. The protein is also found in undifferentiated neuronal progenitor cells and it is thought that this repressor may act as a master negative regulator of neurogenesis [96,97,98]. Alternatively-spliced transcript variants have been described [99]. Expression correlation analyses showed a weak correlation of FAM72A with REST in glioma (Figure 4d).

### 4.5. FAM72 Expression and Long Non-Coding RNAs

Additionally, it has been hypothesized that IGR regulation of the |-SRGAP2–FAM72-| master gene is susceptible to long non-coding RNAs (lncRNAs) [1,5,8]. Long non-coding RNAs (LncRNAs) are of particular interest due to the wide variety of roles they play in gene regulation. LncRNAs have been reported to regulate transcription (via epigenetic mechanisms [100,101]) as well as pluripotency and cellular reprogramming [102] and have been implicated in a variety of diseases, notably cancers of the breast [103], colon [104], stomach [105], lymph [24], and the CNS [106]. 

Recent reports about the oncogenic role of lncRNA revealed interactions between a lncRNA and the centrosomal protein CEP112 as well as the breast cancer type 1 susceptibility protein BRCA1, which resulted in mitotic abnormalities and malignancies [107]. The particular lncRNA, called genomic instability inducing RNA (Ginir), functions normally during embryonic development and is enriched in the brain. The expression of Ginir, along with its partner genomic instability inducing RNA antisense (Giniras), was regulated in a spatio-temporal manner and overexpression of Ginir led to tumorigenesis [107]. This ties in with the role of lncRNA in FAM72 expression. Since FAM72 is also expressed predominantly in NSCs, it is likely that the transcription of the |-SRGAP2–FAM72-| master gene is regulated by a similar pair of lncRNAs on the IGR. FAM72 co-expresses with centrosomal proteins in cancer tissues [12], and it is possible that dysfunction of the lncRNA on the IGR would lead to loss of control over FAM72 expression, thereby leading to cellular proliferation.

### 4.6. Anti-Apoptotic Features of |-SRGAP2–FAM72-| via TP53

Our previous study showed an early anti-apoptotic rescue program activated via the IGR-based expression of the |-Srgap2–Fam72a-| master gene under serum-free stress conditions in rat PC12 cells. Tp53 was thought to influence Fam72 activities in this stress response to rescue cells from apoptosis by driving them into the G0 phase, a possible new anti-apoptotic functional ability of Fam72a [8]. This anti-apoptotic activity of Fam72a was recently consolidated with its highly correlated expression with BIRC5 (Figure 4, Supplementary Materials Figure S2) [108], a member of the family of IAPs that prevent apoptotic cell death [109]. IAP family members usually contain multiple BIR domains, but BIRC5 encodes a protein with only a single BIR domain. The encoded protein also lacks a C-terminus RING finger domain. Along with FAM72A, the FOXM1 protein was also found to be similarly co-regulated with BIRC5 [108]. BIRC5 expression is high in most tumors; however, its usefulness as a prognostic marker is still a controversial issue [110,111].

Although TP53-mediated impact on FAM72 might be indirect, we found a TATA box and a SP1-1 TFBS on the IGR, which could be bound by TP53 with high affinity, thereby eventually affecting FAM72 directly or indirectly by blocking those positions for other TFs (Figure 5) [112,113,114].

On the other hand, TP53 could also bind SP1-1 TFBS on the IGR for transcription regulation by competing with the SP1 protein. To enter G0, downregulation of cell division cycle 25C (CDC25C), another key molecule for cell cycle progression through the G2/M phase [115,116,117], is mediated by TP53 via two independent mechanisms. One of these involves direct binding to the CDC25C promoter [114].

**Figure 7:**
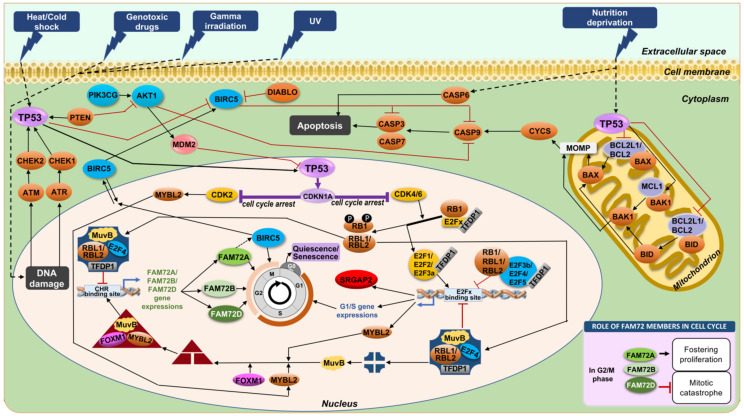
The influence of TP53 on FAM72 paralogs directly regulates the cell cycle in cancer. Upon a stressful DNA-damaging signal (e.g., gamma irradiation), TP53 gets activated to mediate cell arrest in G0 to give the cell quiescence for cell repair or, if impossible, to induce the alternative pathway for apoptosis. TP53-mediated cell cycle arrest is conveyed by CDKN1A (p21) causing inhibition of the cell-cycle promoting the CDK4/6-E2Fx pathway; consequently, the G1/S phase genes remain blocked. The TP53-CDKN1A-CDK4/6 pathway also causes activation of DREAM, which in turn blocks FAM72 expression via the CHR element within the IGR of the |-SRGAP2–FAM72-| master gene.

In another scenario, FAM72 expression was regulated by both DREAM and MMB–FOXM1 complexes under the control of TP53, particularly in cancer cells [75,76]. Through inhibition of cyclin-dependent kinase (CDK) activity by the CDK inhibitor 1A (encoded by CDKN1A), FAM72A and FAM72D were downregulated by TP53 in response to DNA damage via interfering with the DREAM and MMB-FOXM1 complex binding via the CHR BS motifs on the IGR. This prevents the FAM72A and FAM72D expressions, respectively, thus confirming FAM72A and FAM72D as G2/M-phase-promoting cell cycle genes (Figure 5 and Figure 7) [75].

Notably, investigation of TP53 interaction network showed that FAM72A, FAM72B, and FAM72D expressions positively and negatively correlated with TP53 expression in multiple types of cancer under unknown pathways. All FAM72 and TP53 are expressed increasingly in kidney renal papillary-cell carcinoma, but are only highly expressed in FAM72B and TP53 in pancreatic adeno-carcinoma, pheochromocytoma, and paraganglioma. In contrast, the TP53 expression went down, while the three FAM72 were upregulated in lung adeno-carcinoma and prostate adeno-carcinoma [118]. The correlations indicate that all members of the FAM72 family have important roles in tumorigenesis and crossing regulation with the tumor suppressor TP53. 

Taken together, FAM72A, FAM72B, and FAM72D can be regulated by the DREAM complex by interacting with the putative E2Fx BS from cell cycle G0/G1/S phases, which are controlled by the RB-E2Fx complex—a specific E2Fx TF complex for cell cycle gene expressions. This pathway was also regulated through inhibition of CDKN1A coordinated by TP53 (Figure 5 and Figure 7). In summary, all FAM72 (A–D) paralogs and TP53 appear to have strictly correlated expression patterns with a possible crucial functional impact on each other. On the topic of interfering with FAM72 expression in the context of tumor cell proliferation, the tumor suppressor TP53-FAM72 linked pathway could be important as an option to inhibit cancer cell proliferation.

The TP53-CDKN1A pathway also takes away the MYBL2 phosphorylation by CDK2, resulting in the activation of MMB-FOXM1, which in turn, could act on the CHR element on the IGR of |-SRGAP2–FAM72-| master gene for FAM72 activation [119,120]. If the DNA damage is too strong (e.g., causing a mutation in a cancer driver proto-oncogene or even in TP53 itself), G2/M phase genes and FAM72 expression remains at high level fostering cancer cell proliferation [12,75,76,78,81]. AKT1, AK strain transforming serine/threonine kinase 1; ATM, Ataxia-telangiectasia mutated serine/threonine kinase; ATR, Ataxia telangiectasia and Rad3-related serine/threonine kinase; BAK1, BCL2 antagonist/killer 1; BAX, BCL2 associated X; BCL2, B cell lymphoma 2; BCL2L1, BCL2 like 1; BID, Bcl-2 homology 3 interacting domain death agonist; CASP3/6/7/9, Caspase 3/6/7/9; CHEK1/2, Checkpoint kinase 1/2; CYCS, Cytochrome c, somatic; DIABLO, Direct inhibitor of apoptosis binding protein with low pI; MCL1, Myeloid cell leukemia 1; MOMP, Mitochondrial outer membrane permeabilization; PIK3CG, Phosphatidylinositol-4,5-bisphosphate 3-kinase catalytic subunit gamma.

## 5. Methylation of FAM72 in Cancerous Tissues

DNA methylation is a well-studied epigenetic modification and involves the covalent attachment of a methyl group to the 5-carbon residue of cytosine [121]. These attachments usually occur on genomic regions with a high density of CpG nucleotides, called CpG islands, but methylation has also been reported in non-CpG regions [122]. Modifications in DNA methylation have been reported from various disorders including multiple sclerosis, diabetes, multiple human cancers as well as neurological disorders [123,124,125,126,127]. Both hyper- and hypomethylation at the CpG islands have been associated with cancers, and there has been a lot of work to understand the mechanisms regulating this behavior [128,129,130]. 

**Figure 8:**
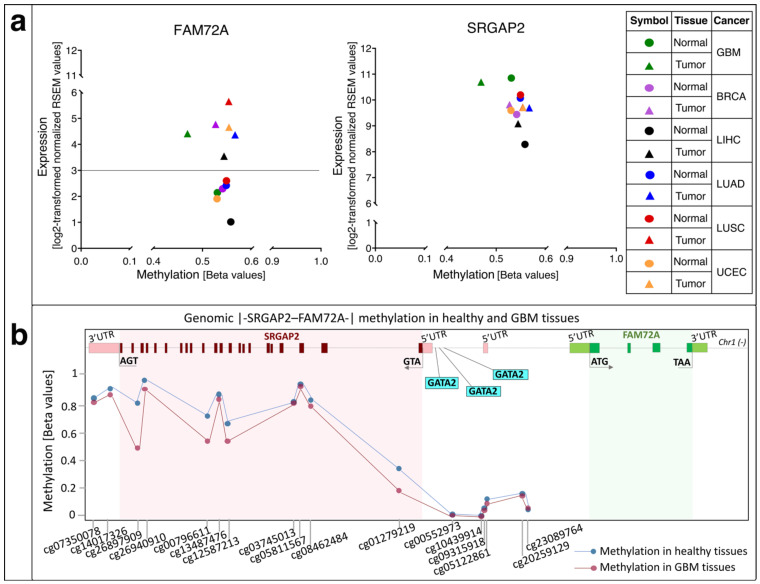
(**a**) Comparison between methylation status and expression levels of FAM72A and SRGAP2 across normal and tumor tissues in GBM, breast, lung, uterine, and liver cancers. Mean methylation beta values were plotted against mean RNA-sequencing by expectation-maximization (RSEM) expression values (log2-transformed normalized RSEM values). Circles indicate normal tissues and triangles indicate cancer tissues. Green symbols indicate GBM data, purple symbols indicate breast invasive carcinoma data, black symbols indicate liver hepatocellular carcinoma data, blue symbols indicate lung adenocarcinoma data, red symbols indicate lung squamous cell carcinoma data, and orange symbols indicate uterine corpus endometrial carcinoma data. In the case of FAM72A, the differences in methylation status between normal and cancer tissues vary among GBM, breast, and liver cancer, where less methylation leads to a two-fold difference in FAM72A expression in cancer tissues. Methylation status between normal and cancer tissues is similar in lung and uterine cancer tissues. In the case of lung tissues, the cancer samples show higher FAM72A expression and a higher methylation status as well, indicating that the FAM72A promoter methylation alone may not be responsible for its increased expression but other factors such as mutations in cancer driver-oncogenes may promote increased FAM72 expression and foster cancer cell proliferation [12,28]. For SRGAP2, the differences in methylation status between normal and cancer tissues is not significant, except eventually for GBM. However, there are no significant changes in SRGAP2 expression in most tissues. Hence, the change of methylation levels within the genomic SRGAP2 area in GBM does not affect SRGAP2 expression. Mean beta values as well as mean mRNA expression values were retrieved from the Wanderer database [136]. BRCA, breast cancer (breast invasive carcinoma); GBM, glioblastoma multiforme; LIHC, liver hepatocellular carcinoma; LUAD, lung adenocarcinoma; LUSC, lung squamous cell carcinoma; UCEC, uterine corpus endometrial carcinoma. (**b**) Investigation of the specific methylation probes on the genomic |-SRGAP2–FAM72A-| master gene in both normal and GBM tissues. Demethylation is described as a decrease in the methylation score of all probes bound to cancer tissue genomes compared to normal tissue genomes. Unfortunately, most probes are focused on the genomic SRGAP2 gene body and IGR area, while no probes could be identified to bind to the genomic FAM72 gene body area. As above, almost no change of SRGAP2 expression level was observed throughout many cancer types including GBM. In contrast, the discovered demethylations may have an impact on regulating the other part of the |-SRGAP2–FAM72-| master gene (i.e., modulating FAM72A expression). The probe information was retrieved from the HumanMethylation450 v1.2 manifest file on the Illumina database (https://support.illumina.com/downloads/infinium_humanmethylation450_product_files.html) (accessed date: 20 December 2020) and aligned the source sequences to genome reference consortium human build 38 patch release 13 (GRCh38.p13) using the BLAST-like alignment tool (BLAT) function from the Integrative Genomics Viewer (IGV) [137,138].

We verified the hypomethylation of FAM72A in GBM, which revealed that expression of FAM72A in GBM could depend on its methylation status [12]. Investigation of the methylation status of FAM72A in non-neuronal tissues revealed that increased expression of FAM72A in lung and uterine cancer tissues appeared to be rather independent of its methylation status (Figure 8a). However, methylation-expression analysis of breast and liver cancer tissues showed an increase in mRNA expression corresponding to a decrease in promoter methylation. The methylation status of the FAM72 promoter thus appears to be important—to a certain extent—in some tissues, namely GBM, breast, and liver cancers, whereas other factors come into play in other non-neuronal tissues. The increased FAM72 expression in non-neuronal tissues is driven by somatic mutations in oncogenes, which would then trigger the signaling cascade for promoting cellular proliferation and fostering tumorigenesis and metastasis [12]. Another factor responsible for increased FAM72 expression could be binding of TFs, which regulate other proliferative genes. We described GATA2 as one of the candidates that could regulate both FAM72 as well as prophase/metaphase cell cycle genes [12]. 

Comparing the corresponding methylation-expression statuses in SRGAP2 revealed that there is no clear difference in its methylation status, which correlates to no or minor changes in its expression status across the same cancer tissues. Indeed, SRGAP2 itself shows no changes in its comparatively higher expression (with FAM72A) with slight differences in promoter methylation of SRGAP2 in breast, liver, lung, and uterine cancers (Figure 8). However, a decrease of methylation (demethylation) on the SRGAP2 gene body in GBM with no changes in its gene expression indicates that this genomic methylation does not affect SRGAP2 gene expression itself, but may rather have an impact on the other coupled gene FAM72A (Figure 8). This also fits with SRGAP2′s established role in neuronal cell differentiation, synaptic maturation as well as neuronal migration [5,8,16,17,26,131]. Usually, SRGAP2 is mobilized to foster neuronal differentiation and synaptic plasticity [4,5,8,16,17,18,19,20,21]. However, in non-neuronal cells, SRGAP2 expression is needed for rearranging the cytoskeleton required for cell-specific locomotion and motility and, if genomic rearranging occurs within the genomic SRGAP2 body, its tumor suppressor function is abolished, and metastasis is induced [26,132,133,134]. Overall, however, it appears that in cancer tissues including GBM [135], the methylation status does not have a major impact on the |-SRGAP2–FAM72-| master gene.

In the brain, the hypothalamus, cerebral cortex, and hippocampus have been reported to be rich sources of oxidized 5-methylcytosine (5mC), thus converting it to 5-hydroxymethylcytosine (5hmC) [139] on enhancers [140]. Demethylation (5hmC) is significantly increased when NSCs and neural progenitor cells (NPGs) differentiate into neurons [141]. This is in contrast to the hypomethylation observed during oncogenesis in FAM72 or the lack of methylation changes observed in SRGAP2, thus confirming that the proliferative and neurogenic mechanisms occur via completely different mechanisms under normal and pathophysiological conditions in the |-SRGAP2–FAM72-| master gene. 5mC-loss/5hmC-gain loci are enriched in active enhancers and motifs for key binding factors involved with neurogenic genes during neurogenesis, as is expected for neurogenic SRGAP2 expression during neuronal differentiation under physiological conditions [142]. The TET1-3 proteins are connected with neural fate decisions [141,142,143,144]. TET2 is a key protein involved in the development and cancer regulating gene expression via oxidization of 5mCs, thereby promoting locus-specific reversal of DNA methylation [145,146]. Thus, TET2 mutations are associated with multiple neurodegenerative diseases [147] and variations of the TET2 gene in either non-coding or coding regions might cause alterations of the homeostasis of key aging-related processes [147]. 

**Figure 9:**
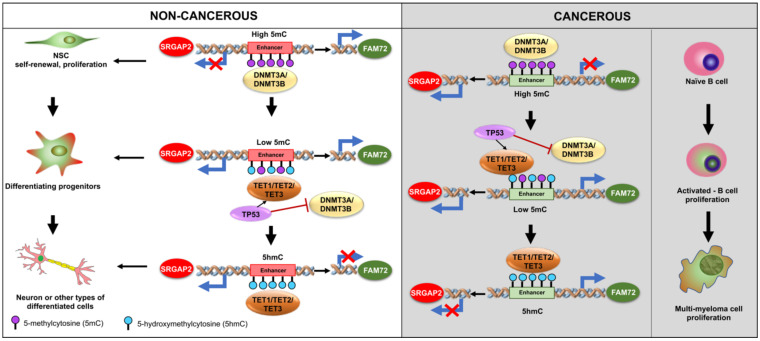
Effects of methylation and demethylation on the expression of the master gene |-SRGAP2–FAM72-|. The epigenetic modifications during neurogenesis can control FAM72 expression for cell fate decision. Dysregulation causes CSC formation and tumorigenesis. In proliferating (non-cancerous) NSCs, demethylation or hypomethylation (such as 5-hydroxymethylcytosine (5hmC) or the loss of the methyl group in the 5-methylcytosine nucleotide (5mC)), were demonstrated to activate neurogenic genes such as SRGAP2 to mediate neural differentiation [142]. As a consequence, FAM72 is deactivated. In cancer, demethylation is crucial for FAM72 activation during CSC proliferation. In the case of glioma genesis, FAM72 is silenced in glia progenitor cells until activated TP53 replaces the methylation factors DNA methyltransferase 3 alpha/beta (DNMT3A/DNMT3B) for demethylation factors TET1/TET2/TET3 so that FAM72 is activated for proliferation and forming GBM cells, which is in line with the genomic hypomethylation of the FAM72 promoter region in our previous study (Figure 8) [12,149,150]. FAM72 expression is activated outside the CNS only under cancerous conditions by mutated protooncogenes and genomic FAM72 demethylation by TET family members to support the proliferation of cancerous cells including multiple myeloma [25,151].

This indicates that TET2 and the 5mC/5hmC mechanism may contribute to |-SRGAP2–FAM72-| master gene activity during neurogenesis (e.g., the 5hmC-gain of neurogenic SRGAP2 during neural differentiation) (Figure 9). Partially differentiated NSCs going into NPGs might be able to concurrently express FAM72 and SRGAP2, thus not resulting in complete loss of 5mC and still gaining some 5hmC. The full 5hmC-gain needed is met only once neurogenic commitment is accomplished, when SRGAP2 is sufficiently expressed and FAM72A expression is completely blocked (e.g., in post-mitotic differentiated neurons). 

Moreover, multiple myeloma [25] and breast cancers [25] showed that FAM72 expression may be dependent on its methylation status. Demethylation (5mC → 5hmC) of FAM72D occurs mainly in intronic enhancers (but outside the IGR area) and could activate FAM72D to maintain mitotic fidelity. This probably also works for the other FAM72 member expressions such as FAM72A, FAM72B, and FAM72C (due to high homology (99%) in amino acid sequences) [25]. As for GBM, Kan et al. could not identify epigenetically affected FAM72, though our data showed a change in the methylation status (Figure 8) [12,135]. 

This observation is also in line with our results obtained in PC12 cells [8]. The hypothesis here is whether GATA2 TFBS (present three times on the IGR of the human |-SRGAP2–FAM72-| master gene, Figure 5) can act as a binding target for this pioneering TF since GATA2 may not directly mediate DNA methylation, but may have an impact on DNA packaging controlled by histone methylation and acetylation [142,148]. GATA TFs can either activate gene expression by synergy with another co-activator (which recruits a histone methyltransferase and/or a histone acetyl transferase) or repress gene expression by cooperating with a co-repressor to recruit a histone demethylase and/or a histone deacetylase [148]. Thus, the GATA2 TFBSs on the IGR may play important roles in the governance of the |-SRGAP2–FAM72-| master gene expression and require further investigation.

## 6. FAM72 and FAM107A in GBM

FAM107A (also known as downregulated in renal cell carcinoma 1 [DRR1]) is a novel unique protein family that exhibits functional similarity with heat shock proteins (HSPs) during the cellular stress response with diverse functions in cancer and the nervous system [152]. Recent evidence indicates that FAM107A is involved in GBM invasion and progression, possibly through the induction of EMT activation by phosphorylation of AKT1 [153]. Accordingly, antibody (against glioblastoma stem cells surface markers glycoprotein cluster of differentiation 44 (CD44) and ephrin receptor A2 (EPHA2)-antisense oligodeoxynucleotides (ASOs) strategy against FAM107A) were established for the treatment of GBM [154]. 

In agreement with FAM107A as a tumor suppressor gene [152,155,156], FAM72A shows a negative expression correlation in GBM (Figure 4b).

## 7. FAM72 and Its Role as a Potential Biomarker in Clinical Cancer Diagnostics

Liquid biopsies carrying circulating tumor-derived material, also called the “tumor circulome,” consist of circulating tumor DNA (ctDNA), circulating tumor RNA (ctRNA), circulating tumor proteins (ctPs), tumor-derived extracellular vesicles (EVs), tumor-educated platelets (TEPs), and circulating tumor cells (CTCs), among others, which have promising diagnostic potential at each stage of cancer [157]. Liquid biopsies have a great potential to overcome existing limitations of tissue biopsies, particularly in light of sampling and analysis of such liquid biological sources, typically blood, for cancer diagnosis, screening, and prognosis. The ‘tumor circulome’ can be directly or indirectly used as a source of cancer biomarkers in liquid biopsies, particularly ctDNA, ctRNA, and ctPs. FAM72, at the ctDNA, ctRNA, and ctP level, could possibly serve as biomarkers for clinical diagnostics of cancer as its expression is usually limited to proliferating NSCs.

## 8. FAM72 and Its Role in Cancer Therapy: Therapeutic Options against Tumorigenic FAM72

Targeting FAM72 could thus be a viable treatment method for several cancer types outside the CNS because knockout of neural-specific FAM72 gene function in non-neuronal tissue may cause spindle assembly defects outside the CNS, followed by cell differentiation, senescence, or death by mitotic catastrophe in all non-neuronal proliferating cancer cells. FAM72 is an attractive target for therapy as it is a proliferative marker expressed during the late G2/M-phase of the cell cycle as well as its low expression in normal non-neuronal tissues [3,12,158], and multiple potential approaches are possible.

### 8.1. Therapeutic Options against Tumorigenic FAM72: RNA Interference (RNAi)

RNAi has emerged as a very effective tool for in vivo selective silencing of gene transcription, and substantial progress has been made in analyzing the therapeutic potential of various RNAi products. There are certain advantages of using RNAi for cancer therapy including the ability to target any gene including FAM72A [4], low dosages, and extended inhibition after a single dose [159]. Recently conducted clinical trials against solid tumors are promising, with the RNAi being delivered via nanoparticles [159,160]. Short hairpin-loop RNAs (shRNAs) have been demonstrated to knockdown FAM72A activity, leading to differentiation in NSCs [4]. This proves the efficacy of the approach in developing therapy against FAM72. Another approach would be to target both small interfering RNAs (siRNAs) as well as telomerase reverse transcriptase and/or MKI67 [161]. Briefly, the authors constructed adenovirus containing siRNAs targeting both MKI67 as well as the telomerase reverse transcriptase. Gene silencing for multiple oncogenes using more than one siRNA have been demonstrated before [162], and the experiment by Fang et al. [161] inhibited renal cancer cells in vitro. An oncolytic vector containing siRNAs targeted toward FAM72A as well as telomerase reverse transcriptases could prove effective without affecting normal cells, especially in non-neuronal tissues. 

Another approach would be the application of ASOs. ASOs are synthetically generated nucleotide sequences, about 12–25 bases long, which can be tailored according to the target sequence of interest. Intracellular binding of the ASO to its target mRNA results in RNAse cleavage, thereby leading to a lack of mRNA translation and protein formation. Currently, there are approximately 90 ongoing clinical cancer trials evaluating treatment with ASOs, with a majority being in phase I [163,164]. Animal models have proved the efficacy in inhibiting tumor formation using MKI67 ASOs, however, issues remain with optimizing dosage and nuclease degradation susceptibility [165,166]. There have been some successes using ASO cancer trials. OT-101, a phosphorothioate ASO designed for the targeted inhibition of human transforming growth factor beta 2 (TGFβ2) mRNA, has proceeded to the phase I/II clinical trial and demonstrated encouraging results [167]. AZD9150, a STAT3-inhibiting ASO, has demonstrated tumor suppressive activity in lung and lymphoma models as well as in a phase1b trial of pretreated lymphoma patients [168,169]. Another group reported that AZD9150 increases drug sensitivity and decreases tumorigenicity in neuroblastomas [170]. Recruitment for AZD9150 trials in colorectal, pancreatic, and lung cancer is ongoing (NCT02983578) [171]. 

Although RNAi-based drug therapeutic trials have been ongoing for some time, it was only in 2018 that the Food and Drug Administration (FDA) approved the first RNAi-based drug ONPATTRO, which is used to treat transthyretin amyloidosis. Due to a better understanding of the clinical development process required for RNAi therapeutics, more candidates are presently in development and trials, especially for cancer [172]. Selection and design of a delivery vector for RNA duplexes targeted toward FAM72 would be critical. Benayoun et al. have already demonstrated RNA silencing for FAM72, utilizing shRNA lentiviral constructs [4]. Alternatively, gRNA delivery via any of the methods above-mentioned could be performed to knockout FAM72.

### 8.2. Therapeutic Options against Tumorigenic FAM72: CRISPR-Cas9

**Figure 10:**
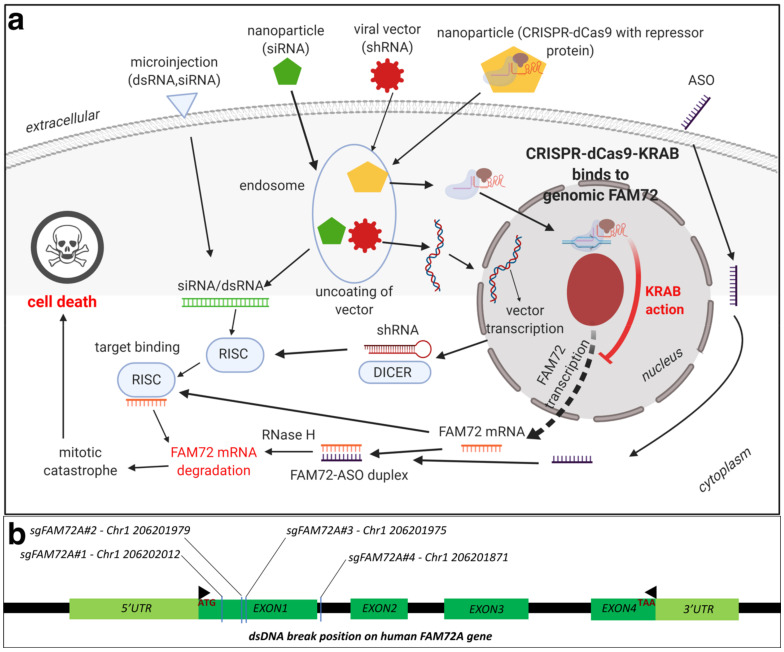
(**a**) Mechanisms of FAM72 knockdown using RNAi and CRISPR for the possible treatment of various types of cancer. Exogenous double-stranded RNA (dsRNA) or siRNA can be delivered via microinjection or lipid nanoparticles. The dsRNA or siRNA is released from the endosome after which it binds to the RNA-induced silencing complex (RISC). This complex then binds to the FAM72 mRNA, leading to the degradation of the whole complex. If shRNA is delivered via plasmid or viral vectors, the RNA is processed in the nucleus and exported into the cytoplasm. The Dicer enzyme processes shRNA into siRNA and then binds it to the RISC, followed by loading onto the target mRNA, and the resulting complex is degraded as before. Alternatively, the CRISPR-dCas9 with a transcriptional repressor protein is delivered via lipid nanoparticles. After entering the endosome, the CRISPR-dCas9 complex is released and it enters the nucleus. The Cas9 nuclease is directed to the target DNA by its bound sgRNA. Following binding of the dCas9 complex with the FAM72 target DNA, the repressor will attach to the transcriptional start site of FAM72, thereby resulting in a knockdown of transcription and thus, prevention of spindle formation causing mitotic catastrophe followed by cell death. ASOs delivered into the cell binding directly to the mRNA transcript, resulting in RNAse degradation. Cas, CRISPR-associated proteins; CRISPR, clustered regularly interspersed short palindromic repeats; dCas9, nuclease deficient Cas9; dsDNA/RNA, double stranded DNA/RNA; KRAB, Kruppel-associated box; sgRNA, single guide RNA; shRNA, short hairpin loop RNA; siRNA, small interfering RNA. (**b**) Double-strand break positions of CRISPR/Cas9 application on the human FAM72A gene. The sgFAM72A#1, sgFAM72A#2, sgFAM72A#3, sgFAM72A#4 have been used to target and break within exon 1 of FAM72A to interrupt FAM72A gene transcription. sgFAM72A, single guide FAM72A: target DNA positions to be recognized and cleaved by the CRISPR/Cas9 system for FAM72A gene expression knockout.

An alternative mechanism to knockout FAM72 in cancer tissues would be to use the clustered regularly interspersed short palindromic repeats (CRISPR)-CRISPR-associated protein (Cas) 9 gene editing tool. Briefly, CRISPR and Cas target foreign viral DNA as part of the adaptive immune system in bacteria [173]. A combination of trans-activating RNA (tracrRNA) and CRISPR targeting RNA (crRNA), together known as small guide RNA (sgRNA or sg FAM72-RNA), guide Cas proteins to the targeted foreign viral (or tumorigenic FAM72) DNA, which is then degraded [174]. The sg FAM72-RNA in combination with the Cas9 protein from Streptococcus pyogenes form the popular CRISPR-Cas9 gene editing tool [175,176,177]. A nuclease deficient Cas9 (dCas9) system combined with a transcriptional repressor protein such as the Kruppel-associated box (KRAB) [178,179] that target the transcription start site for FAM72 would be ideal to knockdown FAM72 in vivo at the site of the tumor [179,180,181,182,183]. Since FAM72 is overexpressed in non-neuronal cancer tissues, such a system would only affect the cancer tissues, leading to greater specificity. The delivery mechanism could be via lipid nanoparticles, similar to siRNA (Figure 10) [184].

### 8.3. Therapeutic Options against Tumorigenic FAM72: Chemotherapy

FAM72 and its paralogs could also be targeted via chemotherapy options using targeted drugs. We conducted an *in silico* binding study to predict potential ligand binding sites on FAM72A [185]. We found potential Zn^2+^ and Fe^3+^ binding sites along with possible binding for the organic compound RSM: (2s)-2-(acetylamino)-N-methyl-4-[(R)-methylsulfinyl] butanamide) [185].

Structure-based drug design (SBDD) is rapidly growing with the development of new technologies (e.g., high-throughput screening, molecular docking, pharmacophore mapping, quantitative structure-activity/property/toxicity relationship (QSAR/QSPR/QSTR), and virtual screening) to interpret, guide, and advance experimental biomedical research to achieve success in anti-cancer drug discovery [186,187,188,189,190]. SBDD methods analyze three-dimensional (3D) structures of macromolecule, typically of proteins or RNA, to identify key sites and interactions, which are important for their specific biological functions [187]. Understanding key sites and interactions can be used to design potential drug candidates that can interfere with essential interactions of the target protein and thus interrupt signaling pathways for survival and progression of cancer cells [187,191]. This requires knowledge of the 3D structure of the drug candidate and how its shape and charge cause it to interact with its biological target, ultimately revealing a therapeutic effect [187,192].

**Figure 11:**
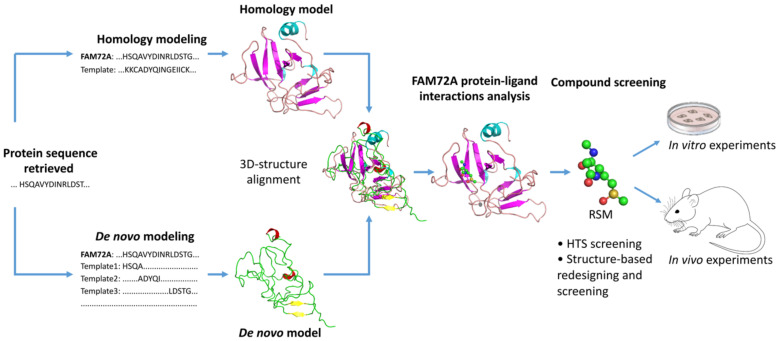
Structure based anti-cancer drug screening for the treatment of FAM72A-mediated cancers. Based on an in silico 3D protein structure model of FAM72A and its ligand-binding sites, the potential hit molecule RSM has been proposed for possible further therapeutic activity evaluations via in vitro and in vivo experiments.

As discussed above in this review, increasing evidence indicates that FAM72 is a potential therapeutic target for the treatment of cancers [1,3,8,158], especially GBM [12] and ACC [28]. In essence, 3D protein structures and understanding ligand–protein interactions of FAM72 represent the key and even obligatory steps in FAM72-targated drug design for the development of a useful treatment for GBM and ACC. There is an urgent need to advance the FAM72-targeted drug design process, and we employed a comprehensive in silico 3D protein determination strategy to determine the 3D protein structure of FAM72A and further identify potential ligand–protein interactions of FAM72A (Figure 11) [185]. An integrated approach combining homology modeling and de novo modeling was applied to obtain a reliable 3D protein structure of FAM72A [185]. In the homology modeling, a homologous template search was performed in various databases (e.g., National Center for Biotechnology Information-Protein Data Bank (NCBI-PDB), Phyre2, 3D-JIGSAW, Swiss Model, and RaptorX) [185]. Additionally, 3D FAM72A protein structure models were also obtained from Phyre2, 3DJIGSAW, Swiss Model, and RaptorX tools. Furthermore, an optimized prediction with the Modeller program [193,194,195] using templates, 1YQ3_D, 4OGC_A, 4OGE_A, 3GA3_A, 3MCA_B, 1I8D_B, 4M0M_A, 2FJA_A, and 3UK7_A (obtained from NCBI-PDB, Phyre2, 3D-JIGSAW, Swiss Model, and RaptorX) revealed that the monomeric 3D FAM72A protein structure, based on the 3GA3_A template, was the most reliable model in terms of stereochemical parameter evaluations (i.e., G-factor, Ramachandran plot analysis, and additional comparative iterative threading assembly refinement (I-TASSER) analysis) [185]. To this end, protein-ligand binding site prediction based on BioLiP protein function database screening (based on COACH, TM-SITE, S-SITE, COFACTOR, and ConCavity methods) [196,197] revealed that FAM72A is a Zn^2+^- or Fe^3+^-containing protein, which could potentially interact with the organic molecule RSM (Figure 11) [185]. Taken together, these data suggest a theoretical view of the 3D structure model of FAM72A and its ligand-binding sites [185]. In our view, these structural and protein–ligand interaction data provide a basis of FAM72A protein ligand-binding sites, which require further investigation using well-defined in vitro and in vivo experiments to confirm the therapeutic activity of the suggested compound as potential leads for drug discovery screenings for the treatment of FAM72A-driven cancers (e.g., GBM and ACC) [185].

## 9. Conclusions

The |-SRGAP2–FAM72-| master gene appears to be a pivotal genomic unit involved in brain development and synaptic plasticity. However, in light of the tissue-specific governance of this master gene, it remains to be seen what differentiates and regulates the expression of the |-FAM72–SRGAP2-| master gene across neuronal and non-neuronal tissues. This knowledge might be crucial for the specific biomedical interference with tumorigenic cell proliferation targeting FAM72.

## Supplementary Materials

The following are available online at https://www.mdpi.com/2072-6694/13/5/1025/s1, Supplementary Figures S1–S21: Correlation of Fam72A mRNA expression with mRNA expression of MKI67, BIRC5 (survivin), E2F1, E2F2, E2F4, E2F6, FAM107A, FOXM1, LIN9, LIN37, LIN54, OLIG2, PAX6, RBBP4, TET2, TFDP1, TFDP2, TP53, SOX2, NRAS, and REST, respectively, across various TCGA human cancer tissues.
