# Supplementary material for: FAM72, Glioblastoma Multiforme (GBM) and Beyond
Source: Cancers (Basel). 2021 Mar 1;13(5):1025. doi: 10.3390/cancers13051025 (PMC7957592; doi:10.3390/cancers13051025)
Supplement: Supplementary file 1 [file cancers-13-01025-s001.pdf]

cancers

# FAM72, Glioblastoma Multiforme (GBM) and Beyond

Nguyen Thi Thanh Ho, Chinmay Satish Rahane, Subrata Pramanik, Pok-Son Kim, Arne Kutzner, Klaus Heese

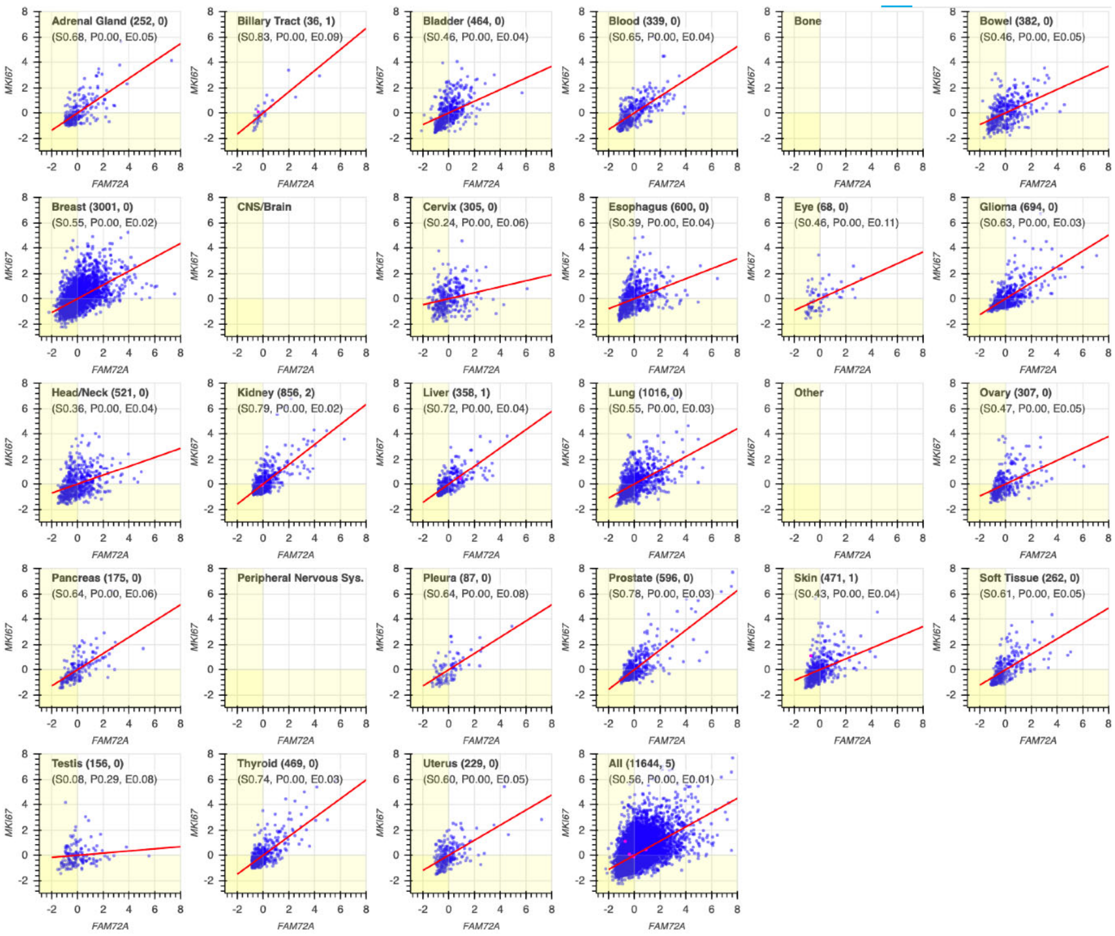


**Figure S1.** Correlation of MKI67 mRNA expression with mRNA expression of FAM72A across various TCGA human cancer tissues. The X-axis represents mRNA expression z-scores for FAM72A, and the Y-axis represents mRNA expression z-scores for MKI67. The blue dots represent mRNA z-scores for each sample in each tissue. FAM72A shows high mRNA expression correlation with MKI67, indicating that both genes are highly expressed in proliferating cells. Sample size, standard error (E), slope (S), and p (P)-values for the graphs are indicated. See also: A cancer tissue-specific FAM72 expression profile defines a novel glioblastoma multiform (GBM) gene-mutation signature. Rahane CS, Kutzner A, Heese K. J Neurooncol. 2019 Jan;141(1):57-70. doi: 10.1007/s11060-018-03029-3.

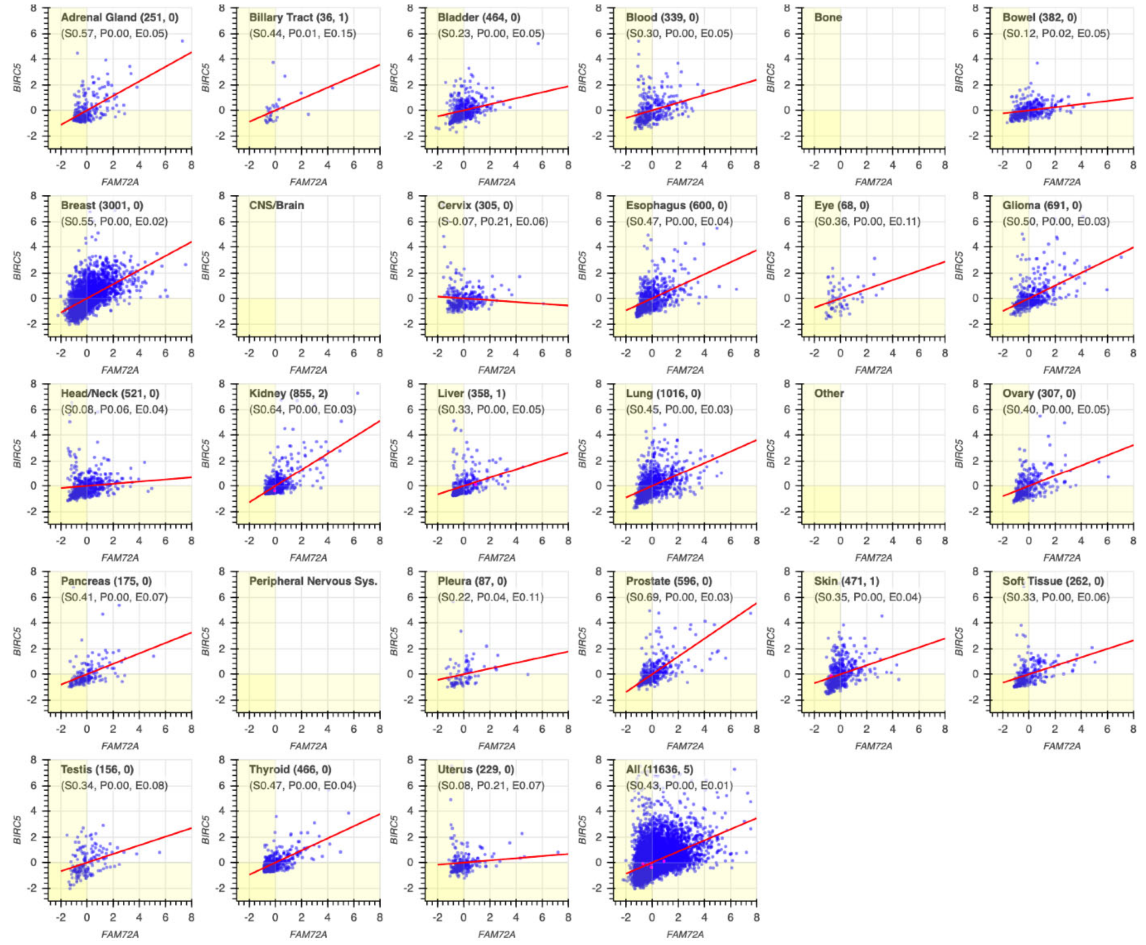


**Figure S2.** Correlation of BIRC5 (survivin) mRNA expression with mRNA expression of FAM72A across various TCGA human cancer tissues. The X-axis represents mRNA expression z-scores for FAM72A, and the Y-axis represents mRNA expression z-scores for BIRC5. The blue dots represent mRNA z-scores for each sample in each tissue. FAM72A shows high mRNA expression correlation with BIRC5, indicating that both genes are highly expressed in proliferating cells. Sample size, standard error (E), slope (S), and p (P)-values for the graphs are indicated. See also:.A cancer tissue-specific FAM72 expression profile defines a novel glioblastoma multiform (GBM) gene-mutation signature. Rahane CS, Kutzner A, Heese K. J Neurooncol. 2019 Jan;141(1):57-70. doi: 10.1007/s11060-018-03029-3.

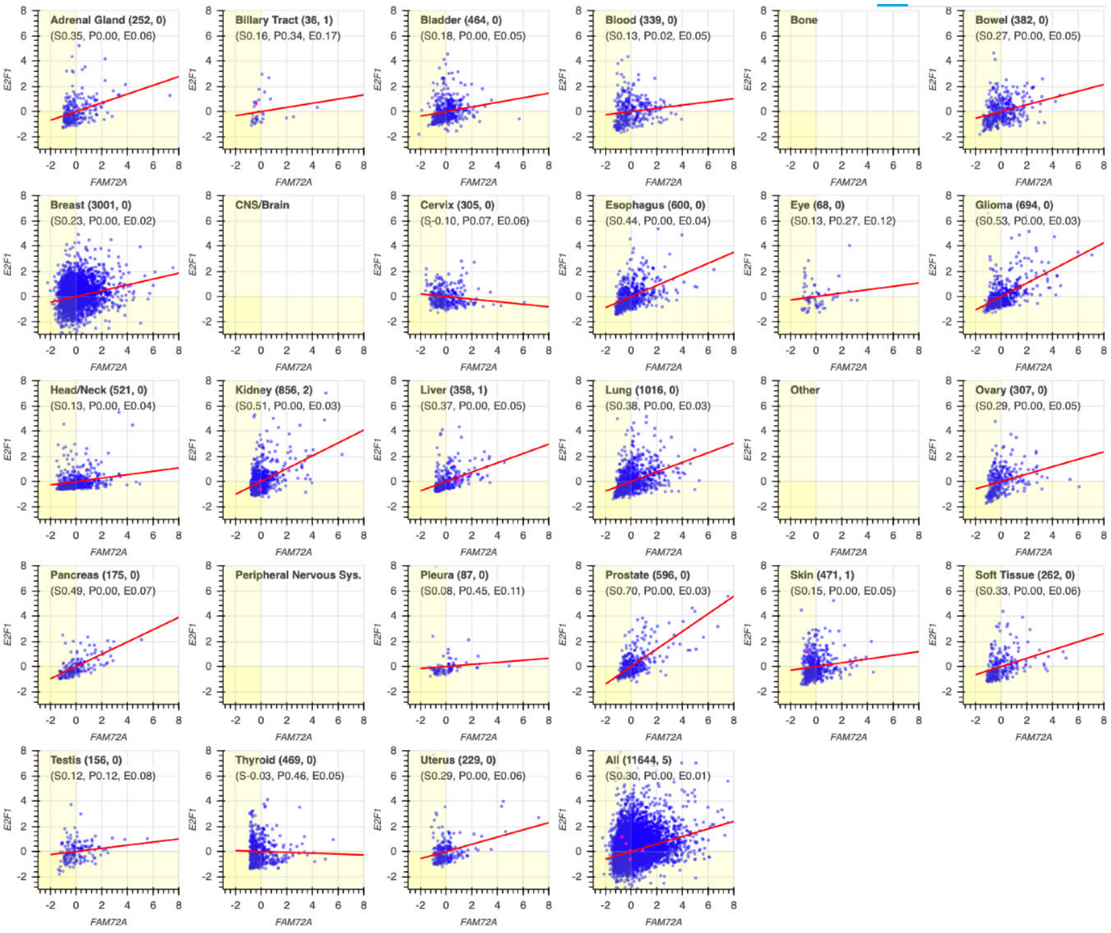


**Figure S3.** Correlation of E2F1 mRNA expression with mRNA expression of FAM72A across various TCGA human cancer tissues. The X-axis represents mRNA expression z-scores for FAM72A, and the Y-axis represents mRNA expression z-scores for E2F1. The blue dots represent mRNA z-scores for each sample in each tissue. FAM72A shows high mRNA expression correlation with E2F1, indicating that both genes are highly expressed in proliferating cells. Sample size, standard error (E), slope (S), and p (P)-values for the graphs are indicated.

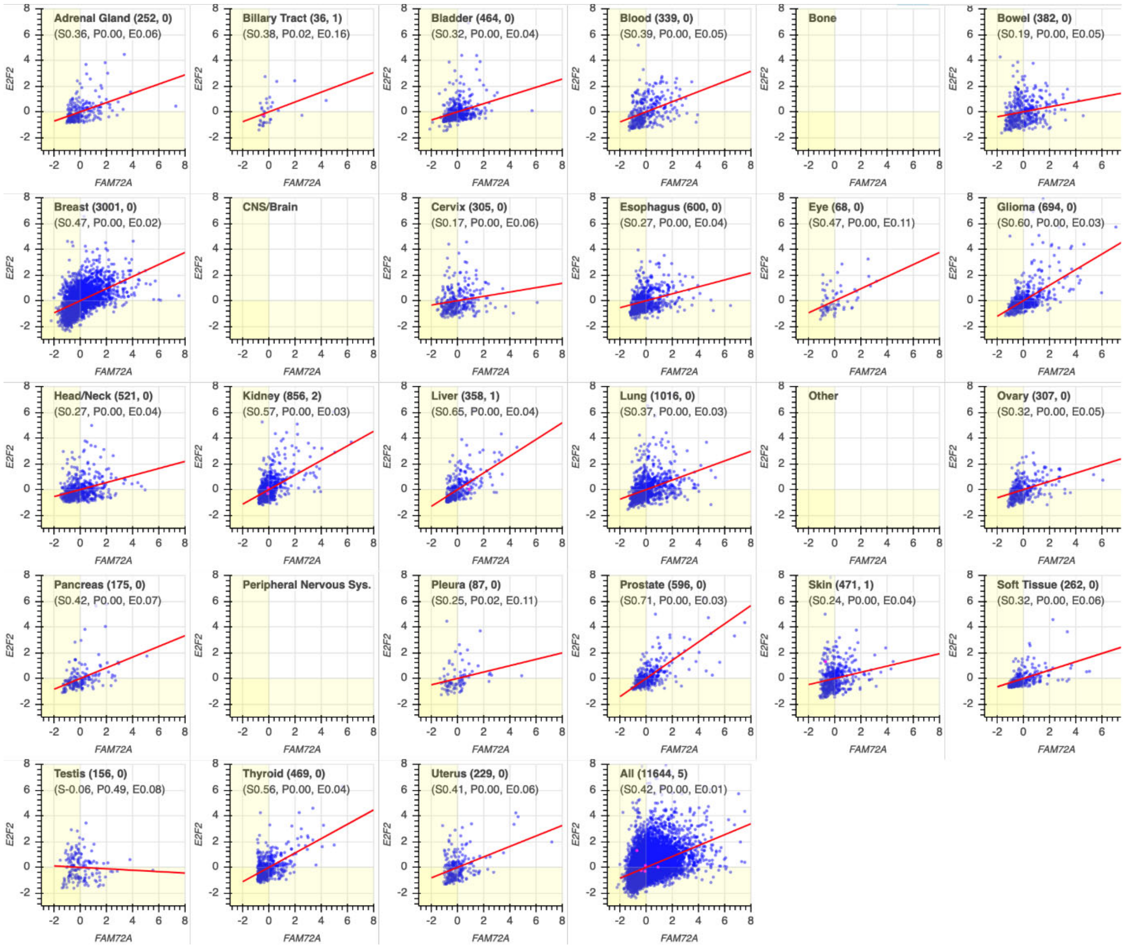


**Figure S4.** Correlation of E2F2 mRNA expression with mRNA expression of FAM72A across various TCGA human cancer tissues. The X-axis represents mRNA expression z-scores for FAM72A, and the Y-axis represents mRNA expression z-scores for E2F2. The blue dots represent mRNA z-scores for each sample in each tissue. FAM72A shows high mRNA expression correlation with E2F2, indicating that both genes are highly expressed in proliferating cells. Sample size, standard error (E), slope (S), and p (P)-values for the graphs are indicated.

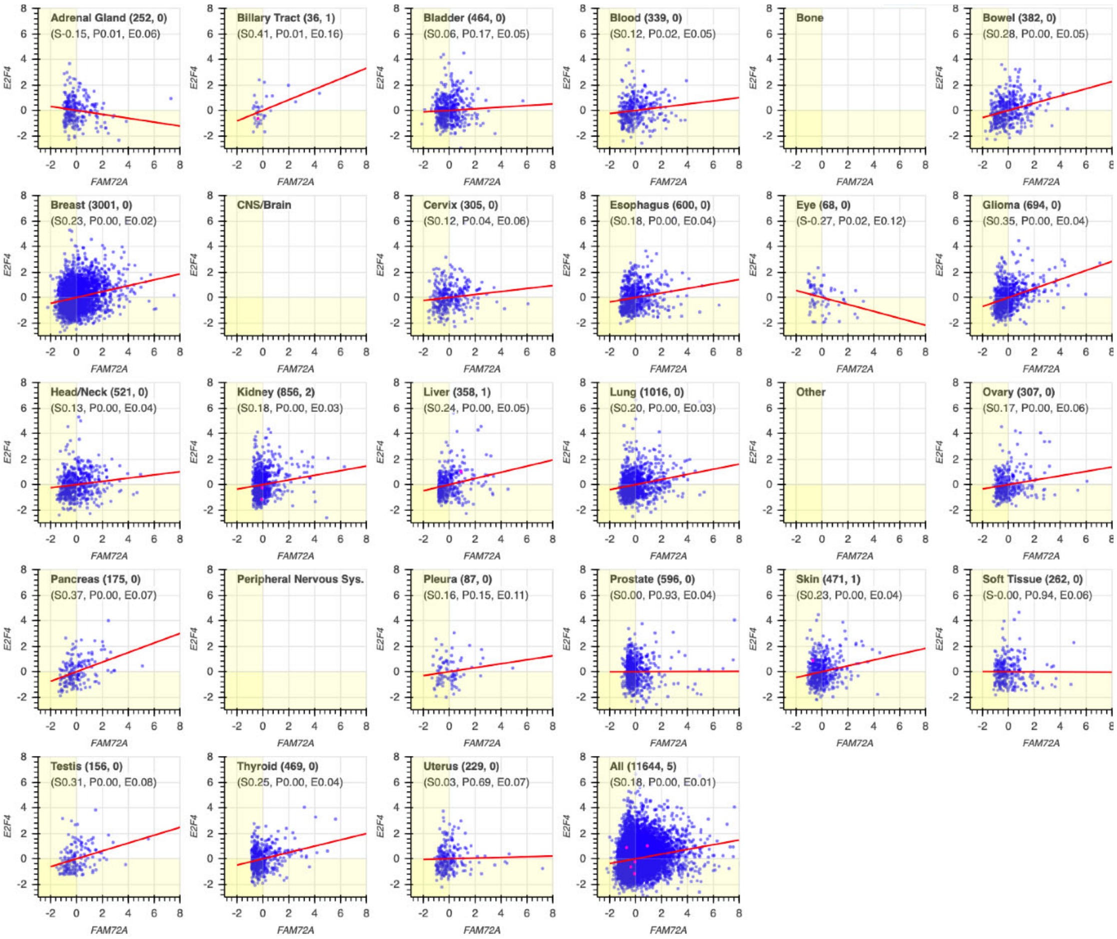


**Figure S5.** Correlation of E2F4 mRNA expression with mRNA expression of FAM72A across various TCGA human cancer tissues. The X-axis represents mRNA expression z-scores for FAM72A, and the Y-axis represents mRNA expression z-scores for E2F4. The blue dots represent mRNA z-scores for each sample in each tissue. FAM72A shows high mRNA expression correlation with E2F4, indicating that both genes are highly expressed in proliferating cells. Sample size, standard error (E), slope (S), and p (P)-values for the graphs are indicated.

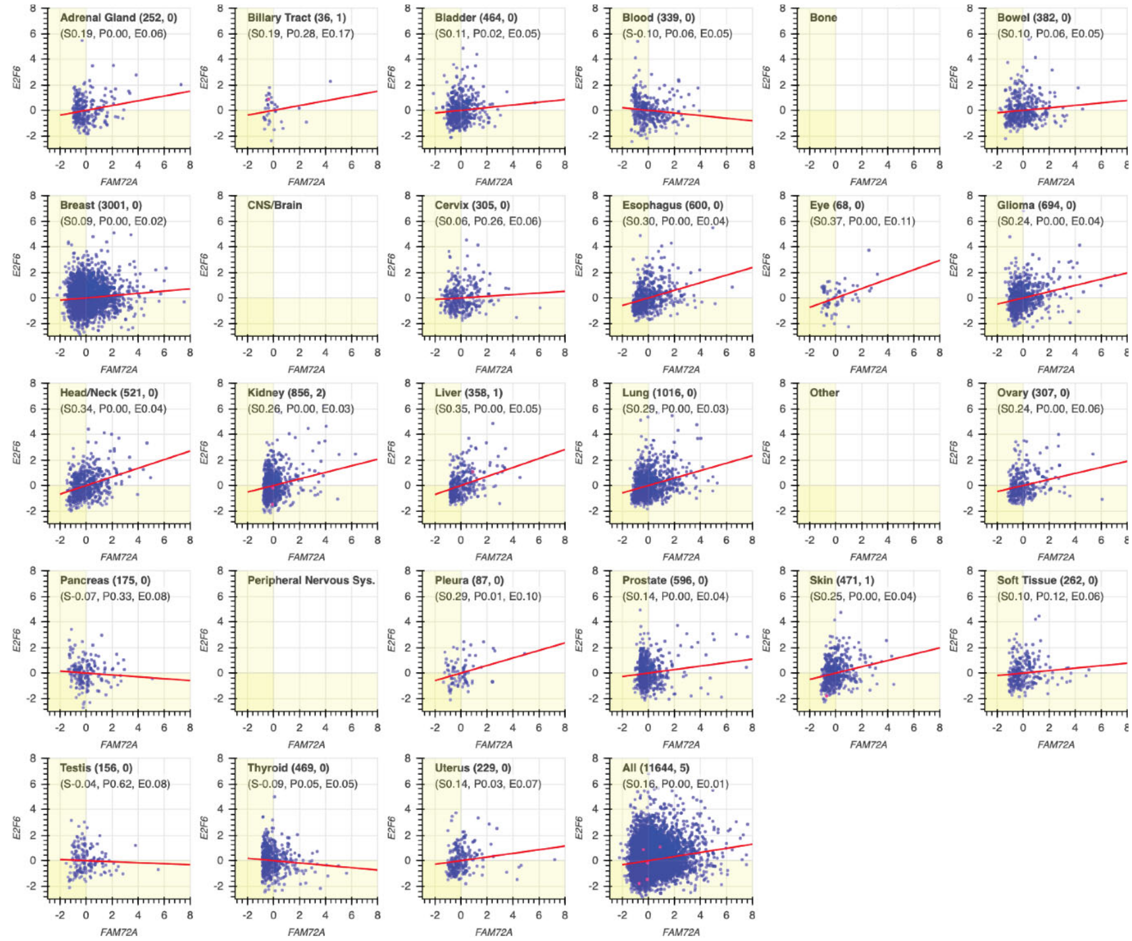

**Figure S6.** Correlation of E2F6 mRNA expression with mRNA expression of FAM72A across various TCGA human cancer tissues. The X-axis represents mRNA expression z-scores for FAM72A, and the Y-axis represents mRNA expression z-scores for E2F6. The blue dots represent mRNA z-scores for each sample in each tissue. FAM72A shows reasonable mRNA expression correlation with E2F6, indicating that both genes are co-expressed in proliferating cells. Sample size, standard error (E), slope (S), and p (P)-values for the graphs are indicated.

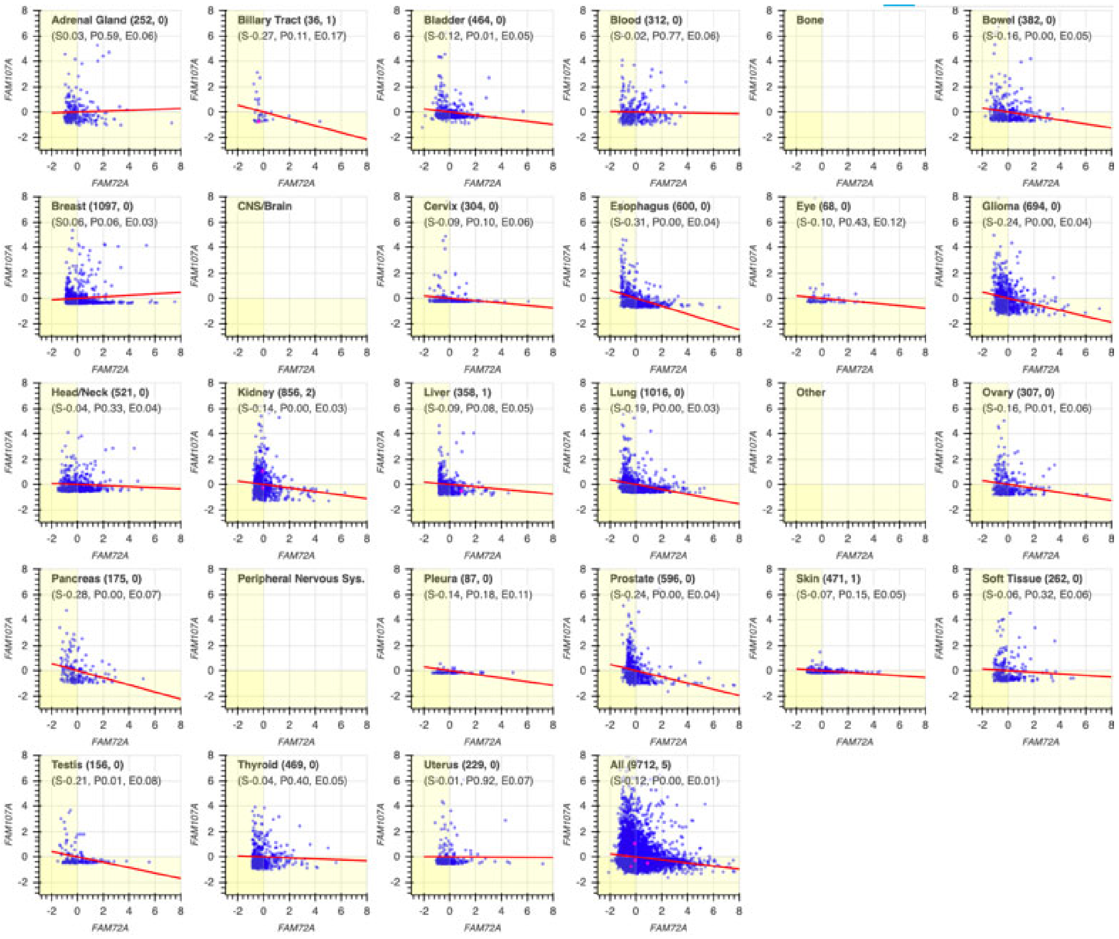


**Figure S7.** Correlation of FAM107A mRNA expression with mRNA expression of FAM72A across various TCGA human cancer tissues. The X-axis represents mRNA expression z-scores for FAM72A, and the Y-axis represents mRNA expression z-scores for FAM107A. The blue dots represent mRNA z-scores for each sample in each tissue. FAM72A shows no mRNA expression correlation with FAM107A, indicating that expression of these genes does not correlate in proliferating cells. Sample size, standard error (E), slope (S), and p (P)-values for the graphs are indicated.

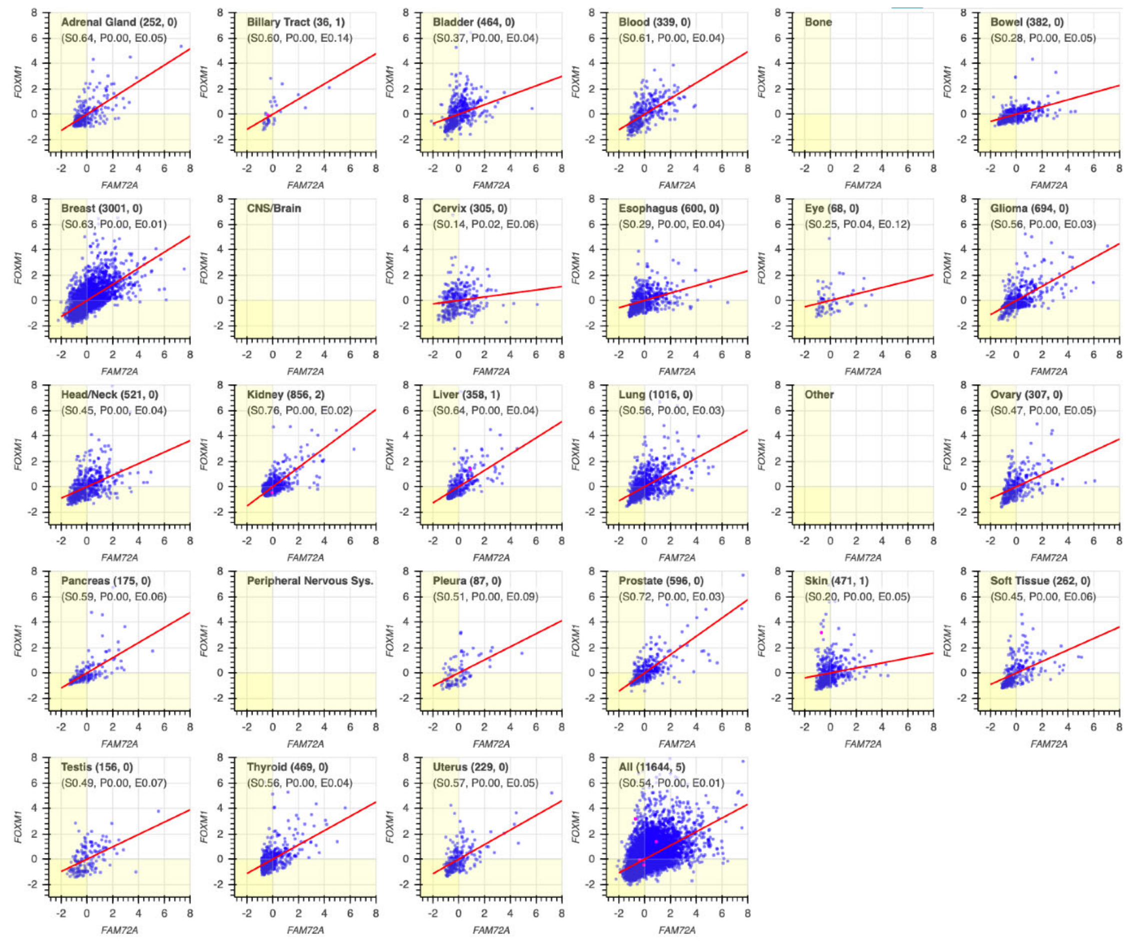


**Figure S8.** Correlation of FOXM1 mRNA expression with mRNA expression of FAM72A across various TCGA human cancer tissues. The X-axis represents mRNA expression z-scores for FAM72A, and the Y-axis represents mRNA expression z-scores for FOXM1. The blue dots represent mRNA z-scores for each sample in each tissue. FAM72A shows high mRNA expression correlation with FOXM1, indicating that both genes are highly expressed in proliferating cells. Sample size, standard error (E), slope (S), and p (P)-values for the graphs are indicated.

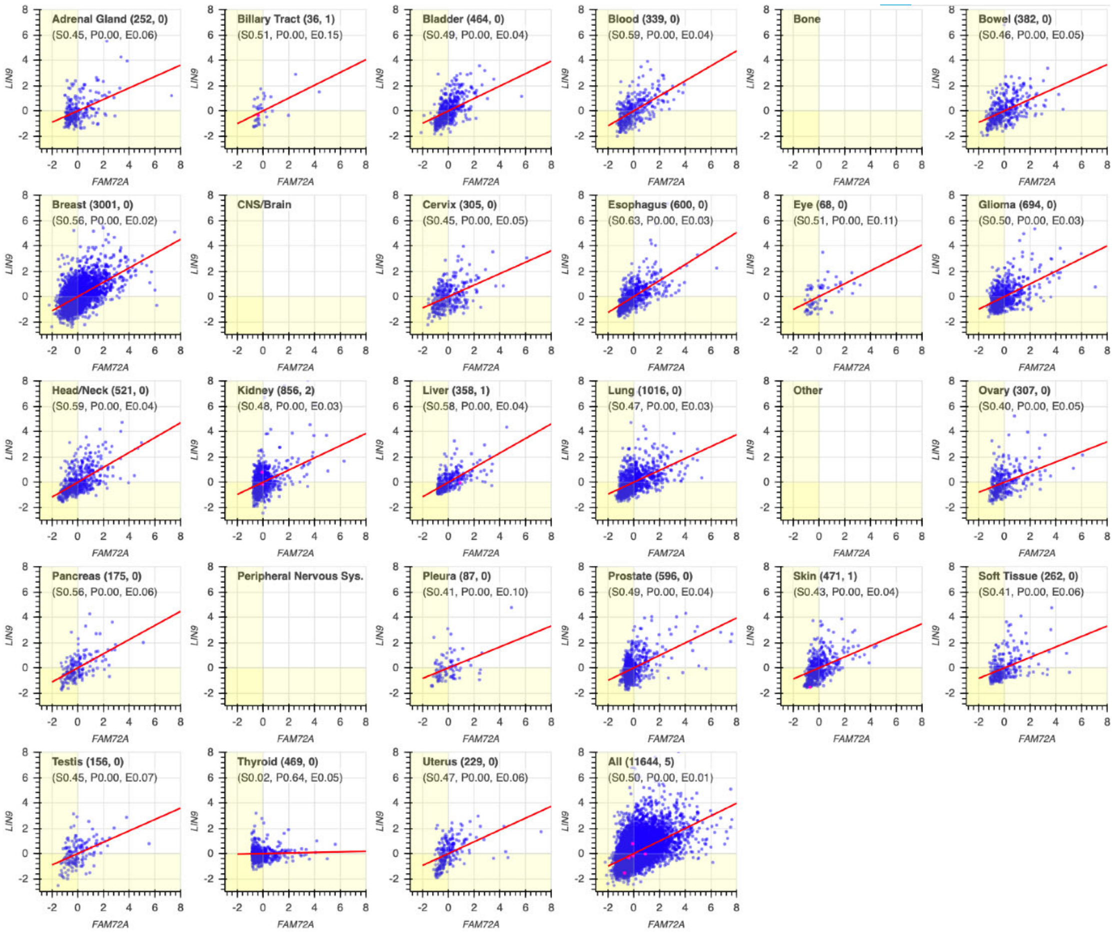


**Figure S9.** Correlation of LIN9 mRNA expression with mRNA expression of FAM72A across various TCGA human cancer tissues. The X-axis represents mRNA expression z-scores for FAM72A, and the Y-axis represents mRNA expression z-scores for LIN9. The blue dots represent mRNA z-scores for each sample in each tissue. FAM72A shows high mRNA expression correlation with LIN9, indicating that both genes are highly expressed in proliferating cells. Sample size, standard error (E), slope (S), and p (P)-values for the graphs are indicated.

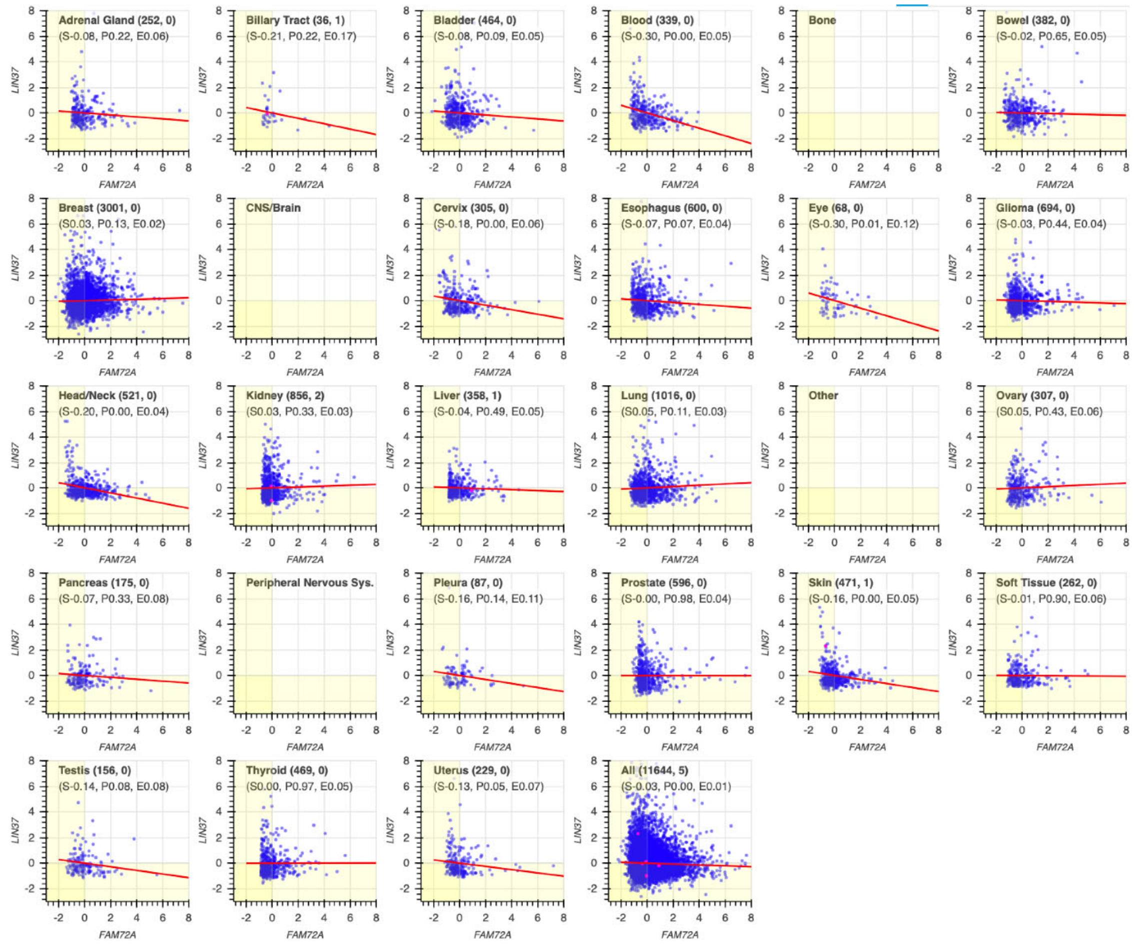


**Figure S10.** Correlation of LIN37 mRNA expression with mRNA expression of FAM72A across various TCGA human cancer tissues. The X-axis represents mRNA expression z-scores for FAM72A, and the Y-axis represents mRNA expression z-scores for LIN37. The blue dots represent mRNA z-scores for each sample in each tissue. FAM72A shows no mRNA expression correlation with LIN37, indicating that expression of these genes does not correlate in proliferating cells. Sample size, standard error (E), slope (S), and p (P)-values for the graphs are indicated.

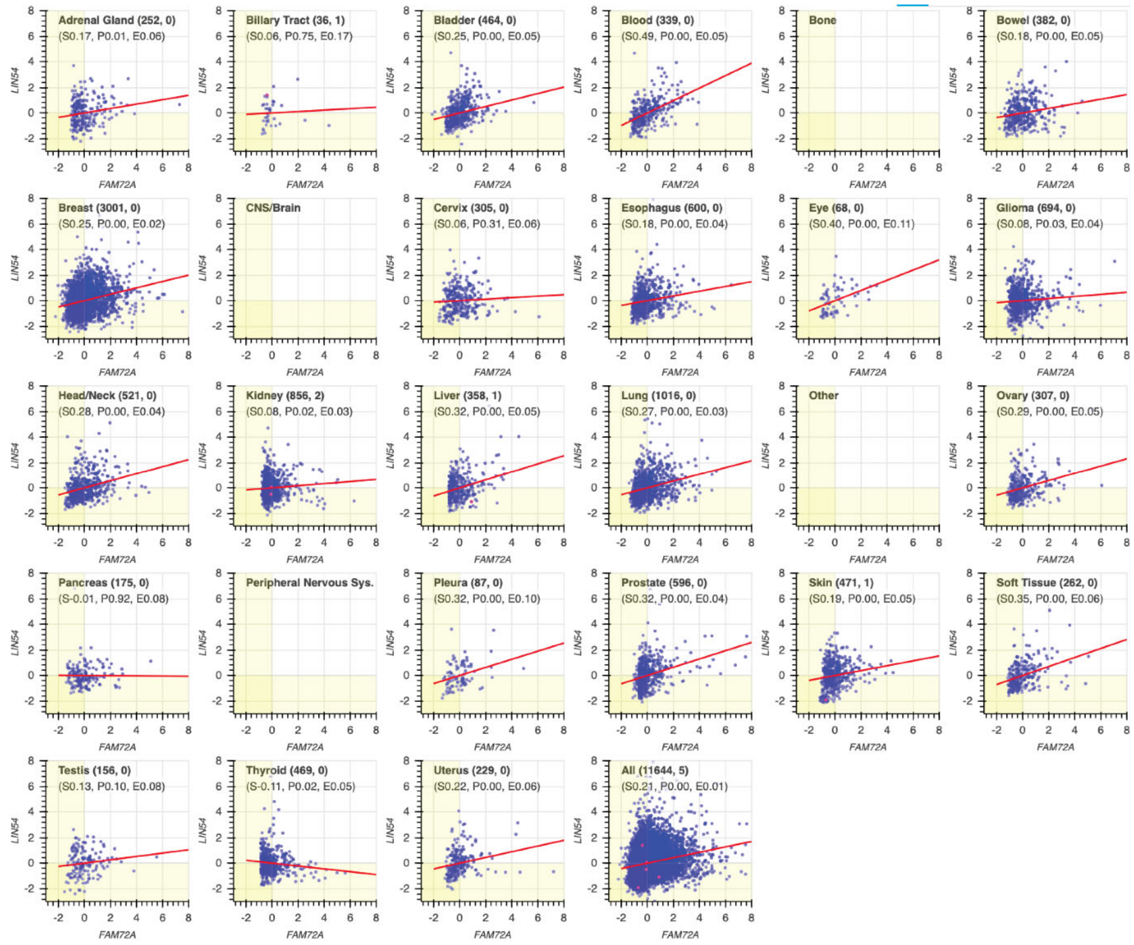


**Figure S11.** Correlation of LIN54 mRNA expression with mRNA expression of FAM72A across various TCGA human cancer tissues. The X-axis represents mRNA expression z-scores for FAM72A, and the Y-axis represents mRNA expression z-scores for LIN54. The blue dots represent mRNA z-scores for each sample in each tissue. FAM72A shows weak mRNA expression correlation with LIN54, indicating that expression of these genes is only weakly correlated in proliferating cells. Sample size, standard error (E), slope (S), and p (P)-values for the graphs are indicated.

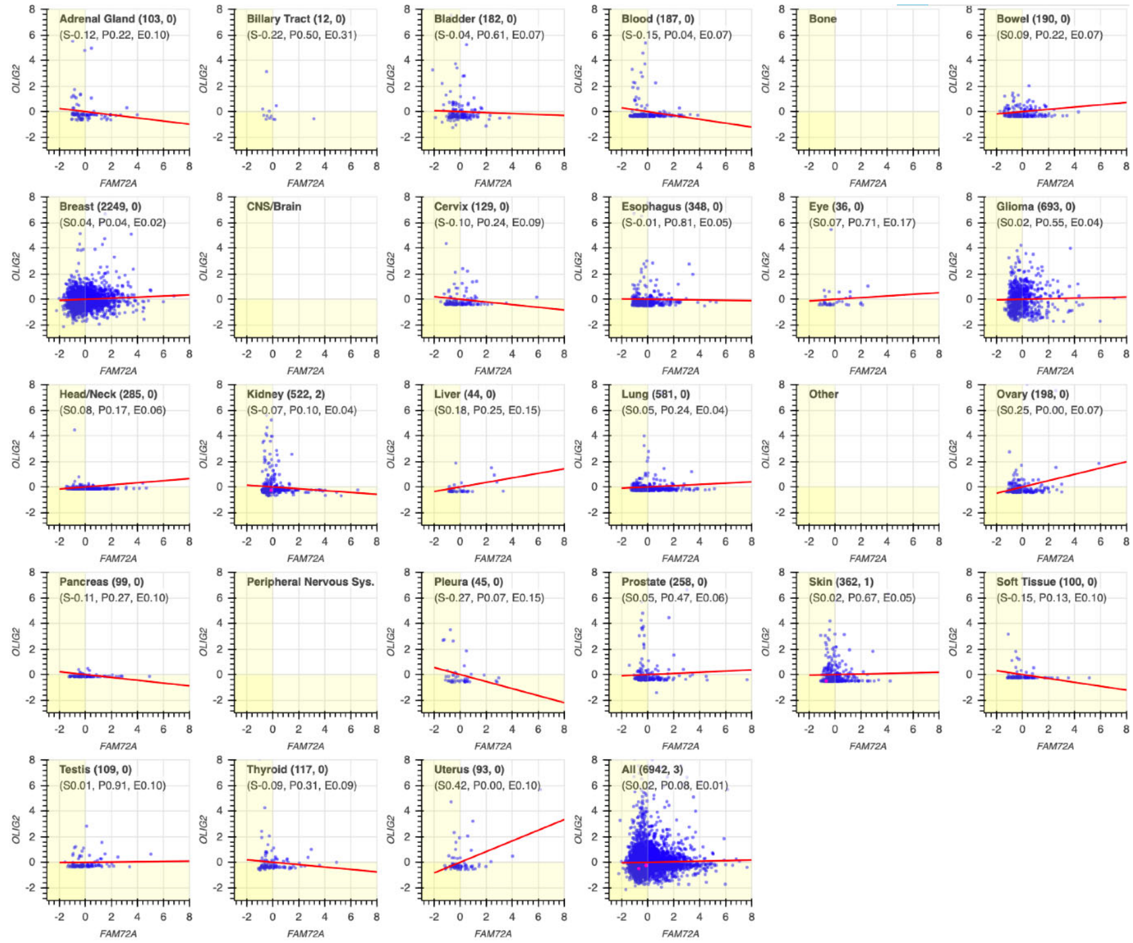


**Figure S12.** Correlation of OLIG2 mRNA expression with mRNA expression of FAM72A across various TCGA human cancer tissues. The X-axis represents mRNA expression z-scores for FAM72A, and the Y-axis represents mRNA expression z-scores for OLIG2. The blue dots represent mRNA z-scores for each sample in each tissue. FAM72A shows no mRNA expression correlation with OLIG2, indicating that expression of these genes does not correlate in proliferating cells. Sample size, standard error (E), slope (S), and p (P)-values for the graphs are indicated.

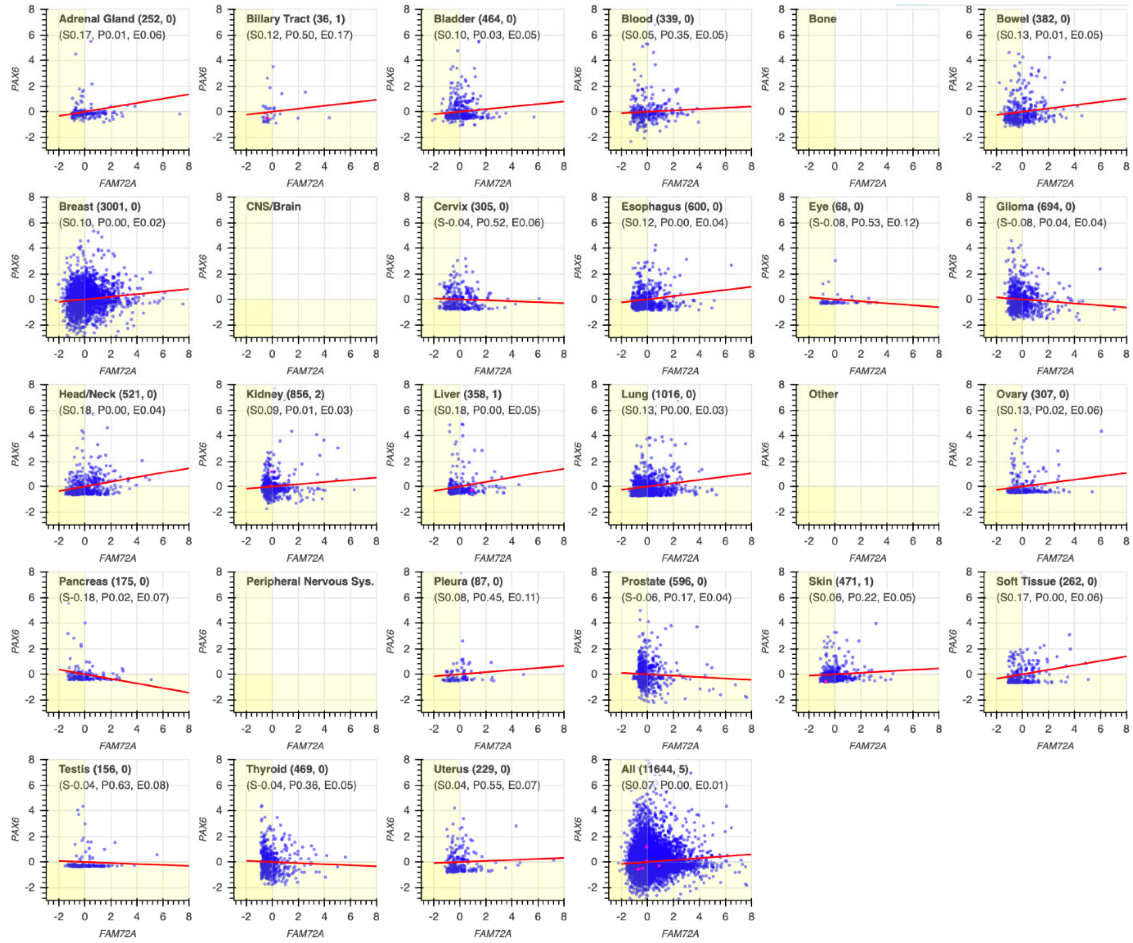


**Figure S13.** Correlation of PAX6 mRNA expression with mRNA expression of FAM72A across various TCGA human cancer tissues. The X-axis represents mRNA expression z-scores for FAM72A, and the Y-axis represents mRNA expression z-scores for PAX6. The blue dots represent mRNA z-scores for each sample in each tissue. FAM72A shows no mRNA expression correlation with PAX6, indicating that expression of these genes does not correlate in proliferating cells. Sample size, standard error (E), slope (S), and p (P)-values for the graphs are indicated.

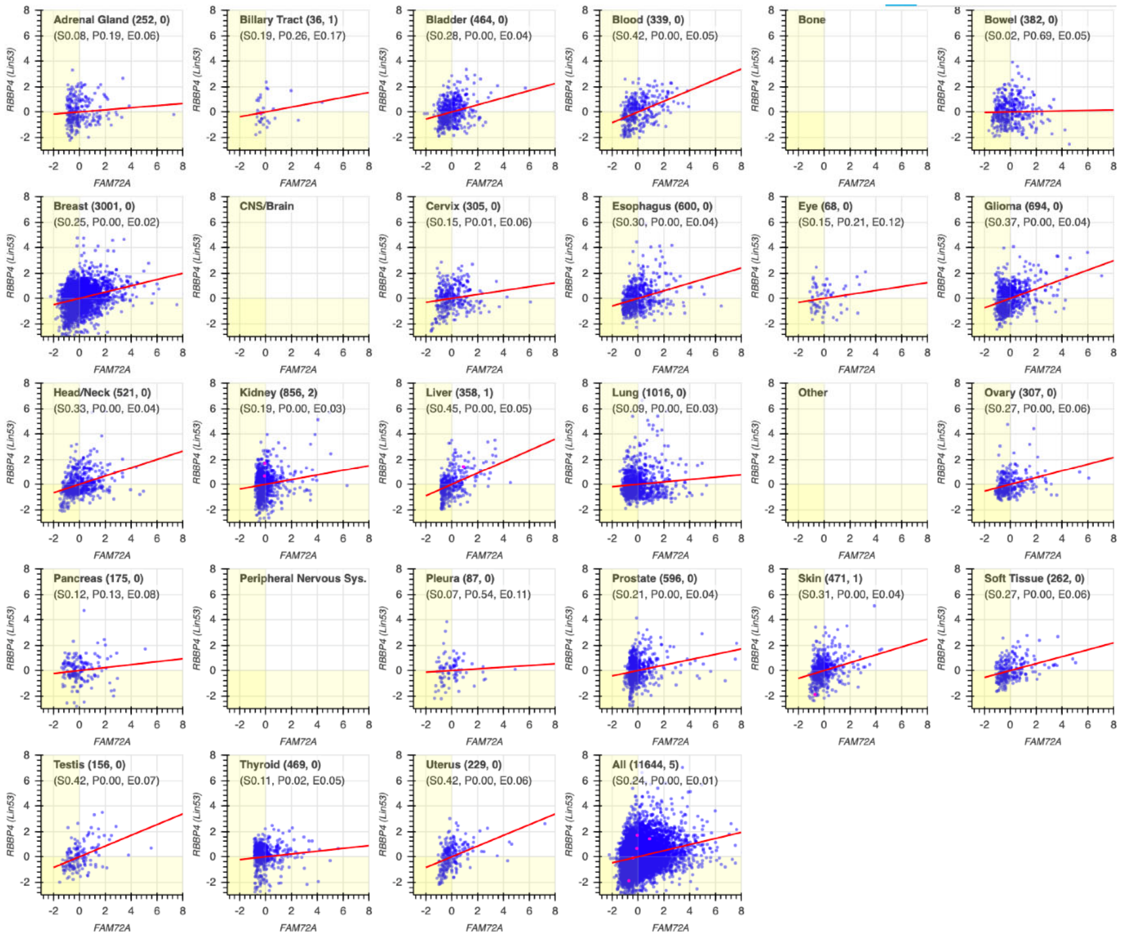


**Figure S14.** Correlation of RBBP4 (LIN53) mRNA expression with mRNA expression of FAM72A across various TCGA human cancer tissues. The X-axis represents mRNA expression z-scores for FAM72A, and the Y-axis represents mRNA expression z-scores for RBBP4 (LIN53). The blue dots represent mRNA z-scores for each sample in each tissue. FAM72A shows high mRNA expression correlation with RBBP4 (LIN53), indicating that both genes are highly expressed in proliferating cells. Sample size, standard error (E), slope (S), and p (P)-values for the graphs are indicated.

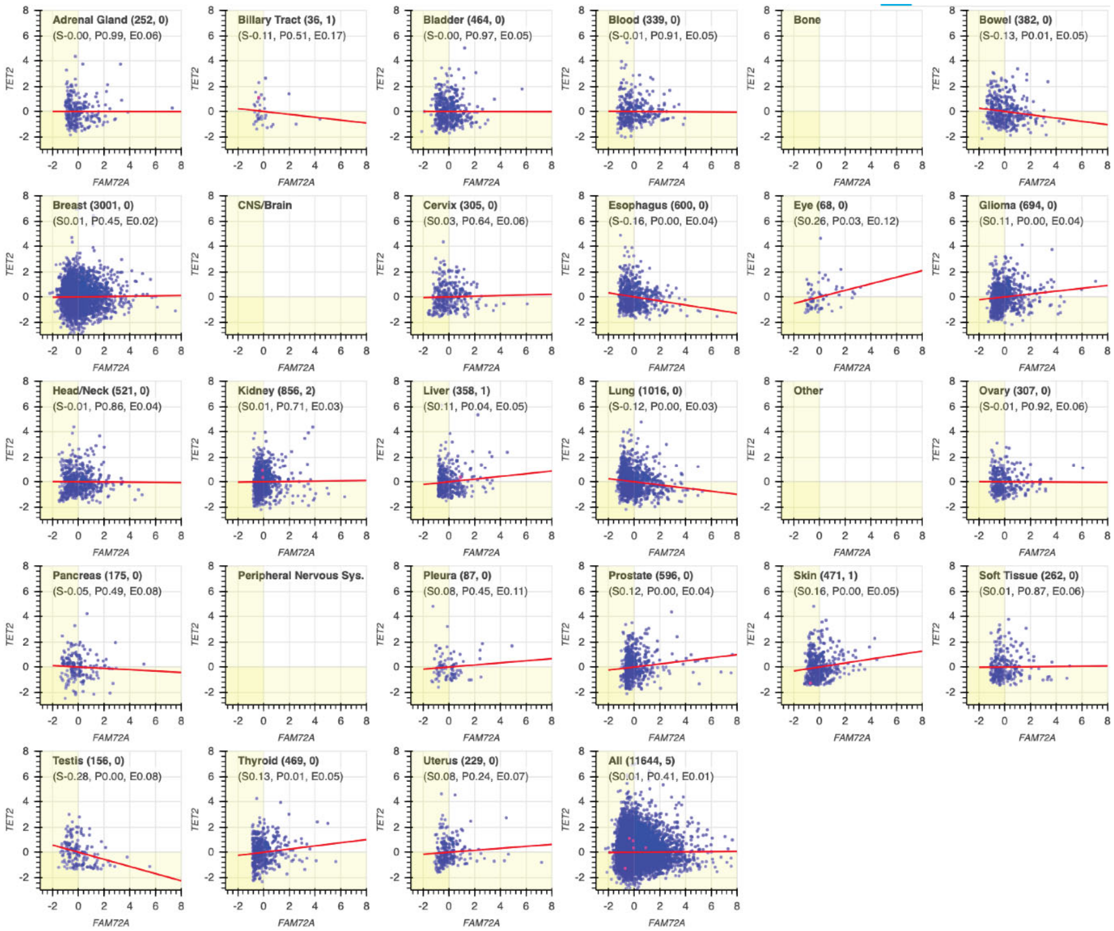


**Figure S15.** Correlation of TET2 mRNA expression with mRNA expression of FAM72A across various TCGA human cancer tissues. The X-axis represents mRNA expression z-scores for FAM72A, and the Y-axis represents mRNA expression z-scores for TET2. The blue dots represent mRNA z-scores for each sample in each tissue. FAM72A shows no mRNA expression correlation with TET2, indicating that expression of these genes does not correlate in proliferating cells. Sample size, standard error (E), slope (S), and p (P)-values for the graphs are indicated.

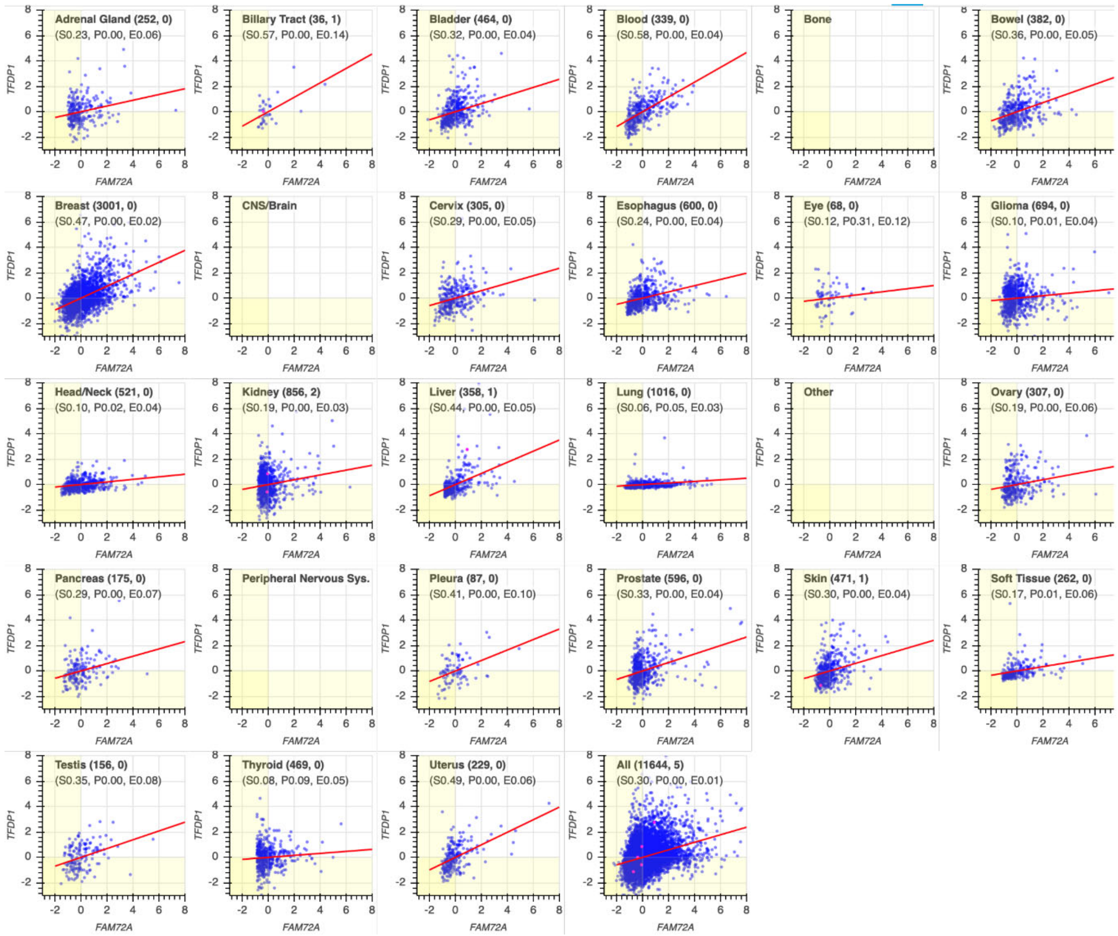

**Figure S16.** Correlation of TFDP1 mRNA expression with mRNA expression of FAM72A across various TCGA human cancer tissues. The X-axis represents mRNA expression z-scores for FAM72A, and the Y-axis represents mRNA expression z-scores for TFDP1. The blue dots represent mRNA z-scores for each sample in each tissue. FAM72A shows weak mRNA expression correlation with TFDP1, indicating that expression of these genes is only weakly correlated in proliferating cells. Sample size, standard error (E), slope (S), and p (P)-values for the graphs are indicated.

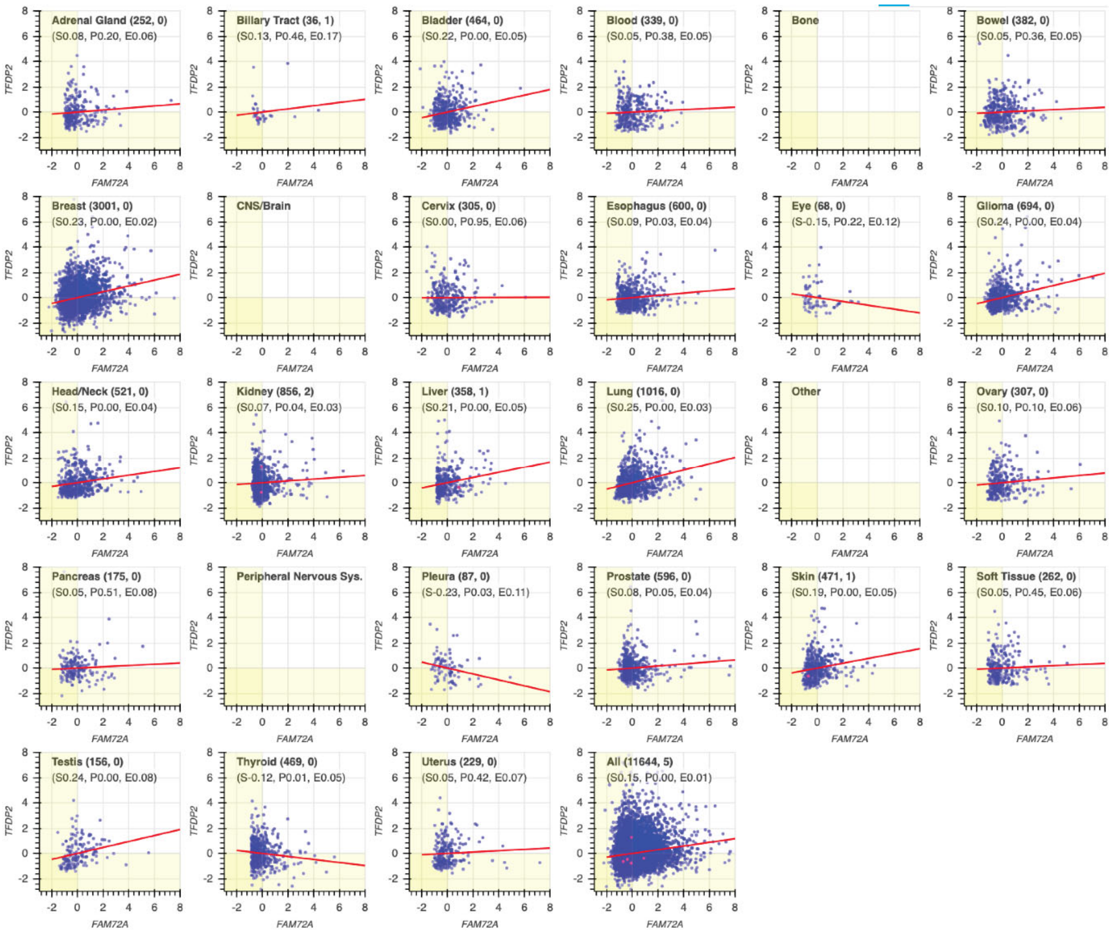


**Figure S17.** Correlation of TFDP2 mRNA expression with mRNA expression of FAM72A across various TCGA human cancer tissues. The X-axis represents mRNA expression z-scores for FAM72A, and the Y-axis represents mRNA expression z-scores for TFDP2. The blue dots represent mRNA z-scores for each sample in each tissue. FAM72A shows weak mRNA expression correlation with TFDP2, indicating that expression of these genes is only weakly correlated in proliferating cells. Sample size, standard error (E), slope (S), and p (P)-values for the graphs are indicated.

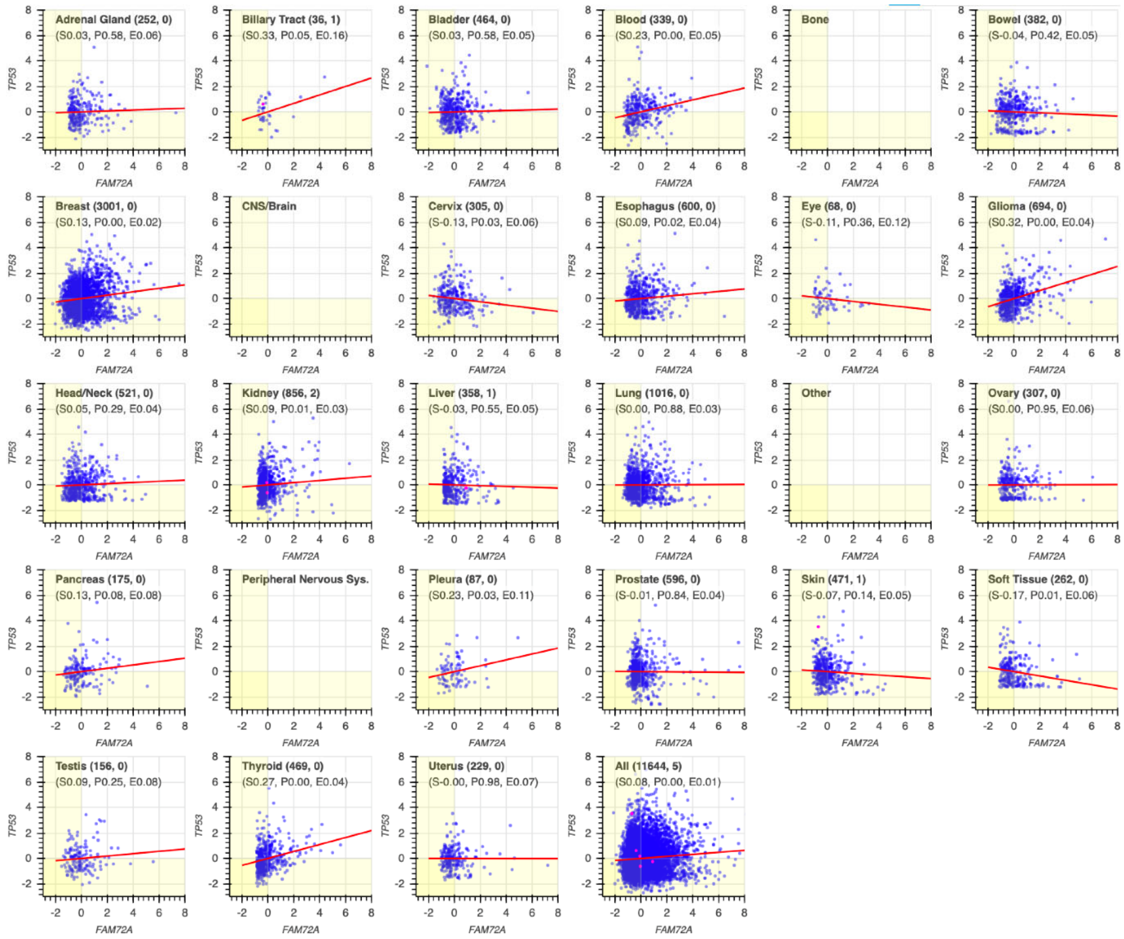


**Figure S18.** Correlation of TP53 mRNA expression with mRNA expression of FAM72A across various TCGA human cancer tissues. The X-axis represents mRNA expression z-scores for FAM72A, and the Y-axis represents mRNA expression z-scores for TP53. The blue dots represent mRNA z-scores for each sample in each tissue. FAM72A shows weak mRNA expression correlation with TP53, indicating that expression of these genes is only weakly correlated in proliferating cells. Sample size, standard error (E), slope (S), and p (P)-values for the graphs are indicated.

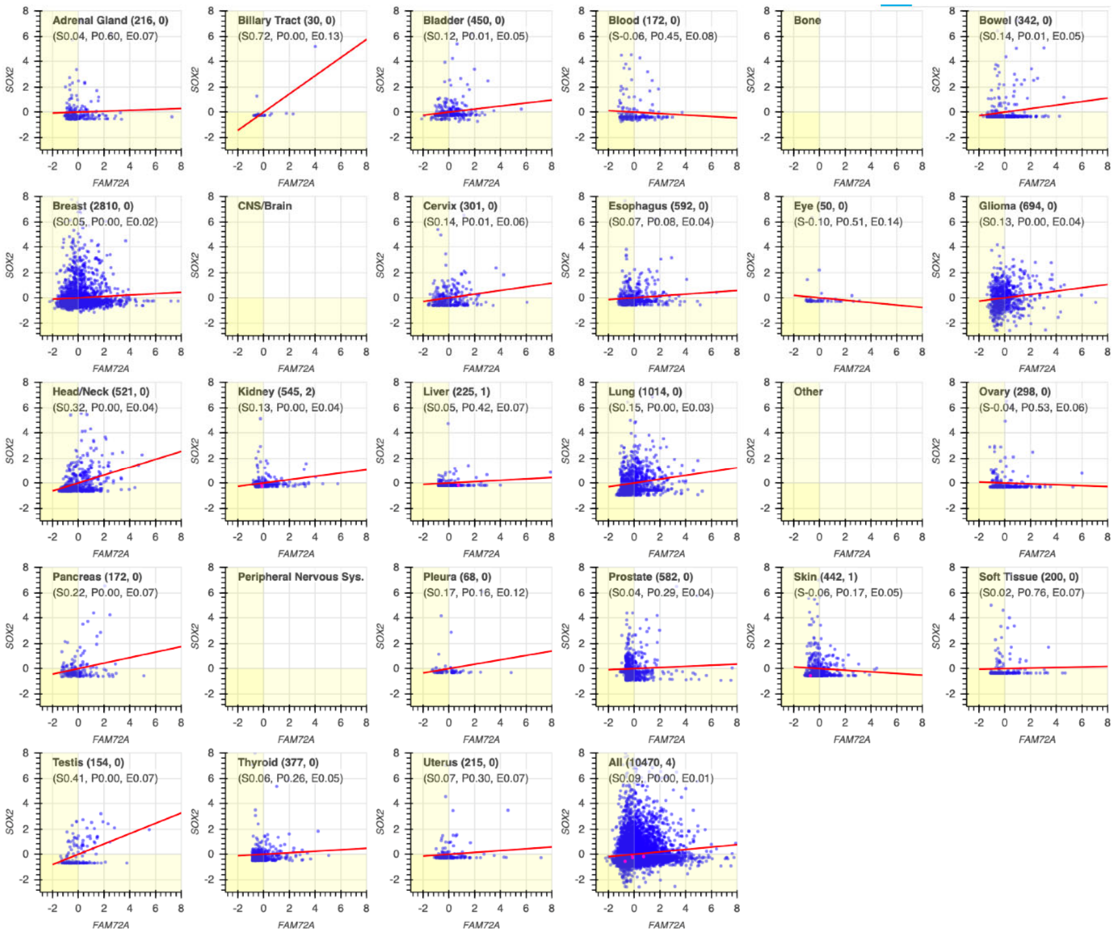


**Figure S19.** Correlation of SOX2 mRNA expression with mRNA expression of FAM72A across various TCGA human cancer tissues. The X-axis represents mRNA expression z-scores for FAM72A, and the Y-axis represents mRNA expression z-scores for SOX2. The blue dots represent mRNA z-scores for each sample in each tissue. FAM72A shows weak mRNA expression correlation with SOX2, indicating that expression of these genes is only weakly correlated in proliferating cells. Sample size, standard error (E), slope (S), and p (P)-values for the graphs are indicated.

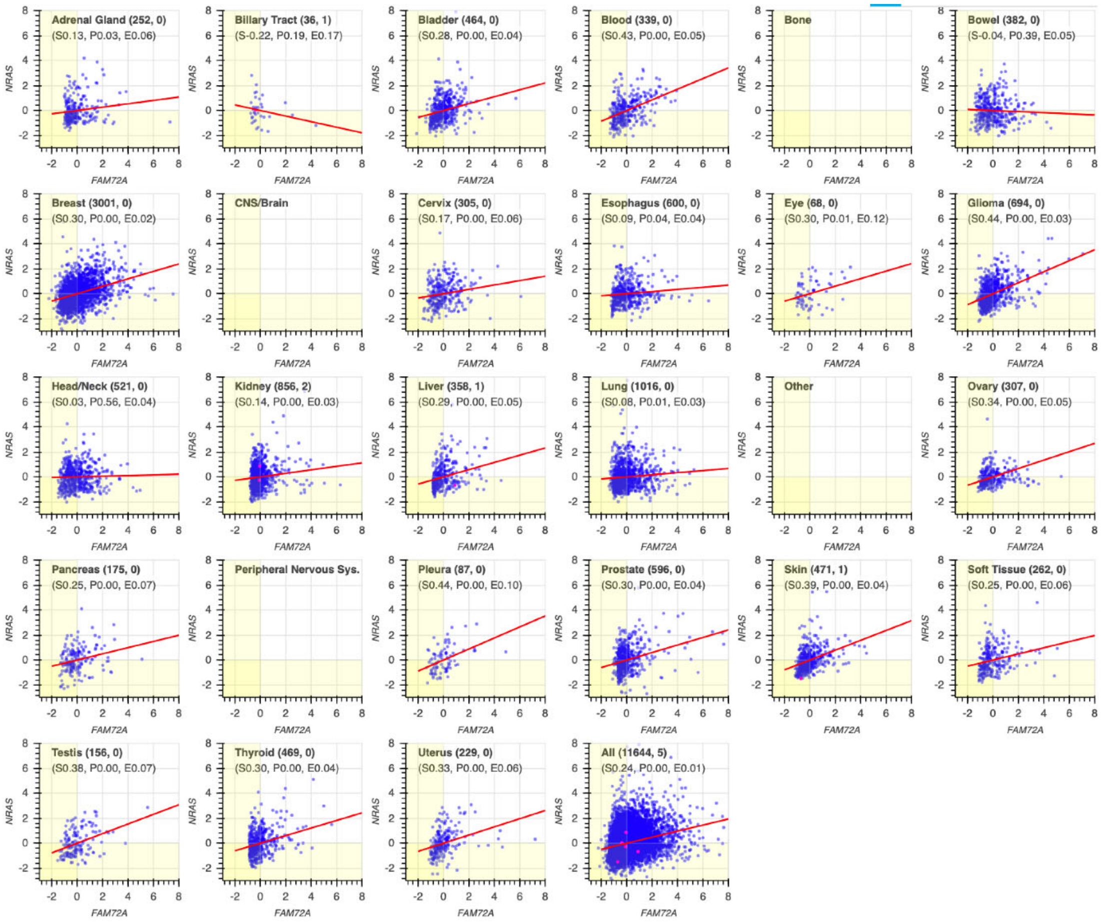


**Figure S20.** Correlation of NRAS (on Chr 1) mRNA expression with mRNA expression of FAM72A (Chr 1) across various TCGA human cancer tissues. The X-axis represents mRNA expression z-scores for FAM72A, and the Y-axis represents mRNA expression z-scores for NRAS. The blue dots represent mRNA z-scores for each sample in each tissue. FAM72A shows weak mRNA expression correlation with NRAS, indicating that expression of these genes is only weakly correlated in proliferating cells. Sample size, standard error (E), slope (S), and p (P)-values for the graphs are indicated.

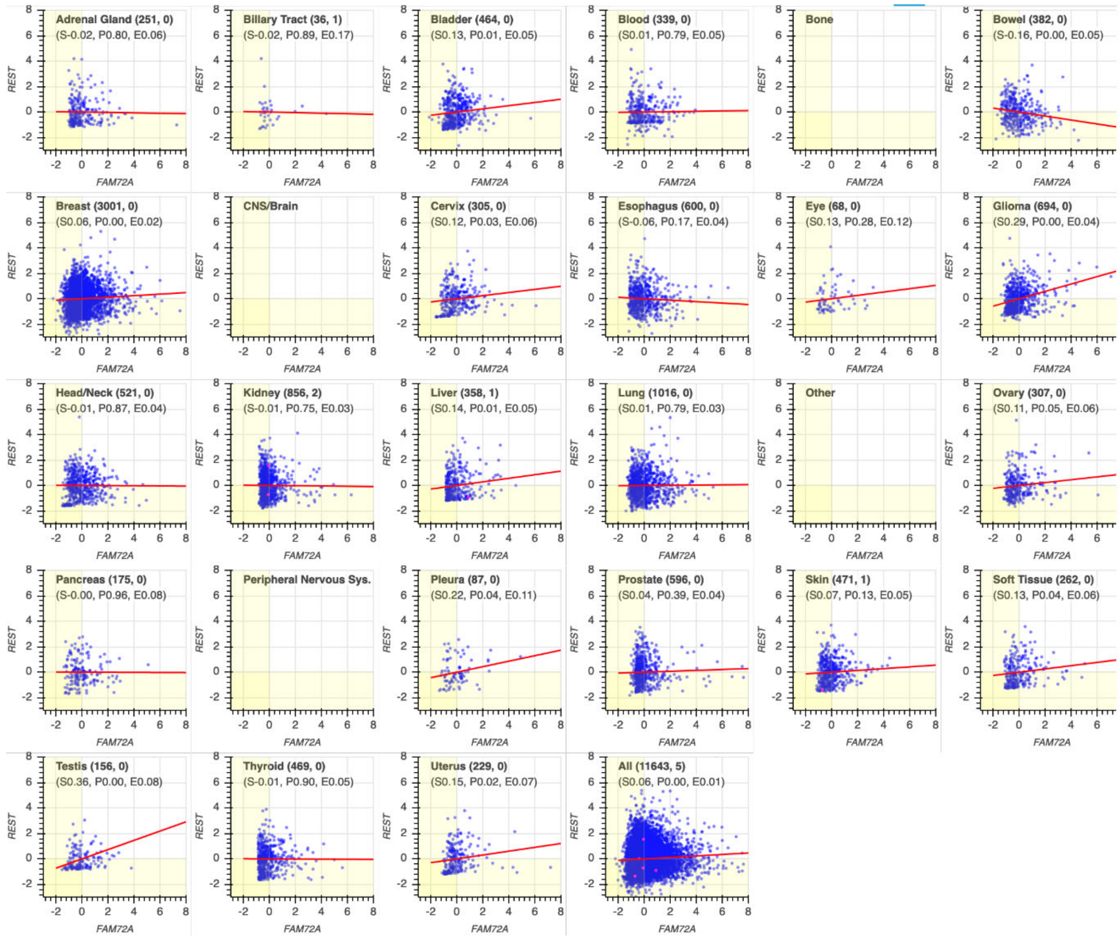


**Figure S21.** Correlation of REST mRNA expression with mRNA expression of FAM72A across various TCGA human cancer tissues. The X-axis represents mRNA expression z-scores for FAM72A, and the Y-axis represents mRNA expression z-scores for REST. The blue dots represent mRNA z-scores for each sample in each tissue. FAM72A shows weak mRNA expression correlation with REST, indicating that expression of these genes is only weakly correlated in proliferating cells. Sample size, standard error (E), slope (S), and p (P)-values for the graphs are indicated.
